# Transcription factor Nrf1 regulates proteotoxic stress-induced autophagy

**DOI:** 10.1083/jcb.202306150

**Published:** 2024-04-24

**Authors:** Madison A. Ward, Janakiram R. Vangala, Hatem Elif Kamber Kaya, Holly A. Byers, Nayyerehalsadat Hosseini, Antonio Diaz, Ana Maria Cuervo, Susmita Kaushik, Senthil K. Radhakrishnan

**Affiliations:** 1 Department of Pathology, Virginia Commonwealth University, Richmond, VA, USA; 2 Massey Comprehensive Cancer Center, Virginia Commonwealth University, Richmond, VA, USA; 3 Department of Developmental and Molecular Biology, Albert Einstein College of Medicine, Bronx, NY, USA; 4 Institute for Aging Research, Albert Einstein College of Medicine, Bronx, NY, USA

## Abstract

Cells exposed to proteotoxic stress invoke adaptive responses aimed at restoring proteostasis. Our previous studies have established a firm role for the transcription factor Nuclear factor-erythroid derived-2-related factor-1 (Nrf1) in responding to proteotoxic stress elicited by inhibition of cellular proteasome. Following proteasome inhibition, Nrf1 mediates new proteasome synthesis, thus enabling the cells to mitigate the proteotoxic stress. Here, we report that under similar circumstances, multiple components of the autophagy–lysosomal pathway (ALP) were transcriptionally upregulated in an Nrf1-dependent fashion, thus providing the cells with an additional route to cope with proteasome insufficiency. In response to proteasome inhibitors, Nrf1-deficient cells displayed profound defects in invoking autophagy and clearance of aggresomes. This phenomenon was also recapitulated in NGLY1 knockout cells, where Nrf1 is known to be non-functional. Conversely, overexpression of Nrf1 induced ALP genes and endowed the cells with an increased capacity to clear aggresomes. Overall, our results significantly expand the role of Nrf1 in shaping the cellular response to proteotoxic stress.

## Teaser

Ward et al. demonstrate that in addition to its well-established role as a positive regulator of proteasome genes, the transcription factor Nrf1/NFE2L1 can also induce autophagy–lysosomal pathway (ALP) genes and drive autophagic flux in response to proteotoxic stress.

## Introduction

Effective destruction and recycling of misfolded, defective, and aggregated proteins is a key step in proteostasis and is essential for cellular survival (Klaips et al., 2018; Lindquist and Kelly, 2011). This step is facilitated by the ubiquitin-proteasome system (UPS) and the autophagy–lysosomal pathway (ALP), the two major proteolytic pathways in the cell (Pohl and Dikic, 2019).

The central player in the UPS is the 26S proteasome, a multisubunit proteolytic complex that recognizes and degrades ubiquitylated substrates (Finley and Prado, 2020). Within the 26S proteasome, the actual process of protein degradation is achieved in the 20S catalytic core that harbors chymotrypsin-like, trypsin-like, and caspase-like activities (Kisselev and Goldberg, 2005). Often, the barrel-shaped 20S core subunit is capped on one or both ends by a 19S regulatory particle that aids in substrate deubiquitylation and unfolding, both of which are essential steps for proteasome-mediated degradation (Bard et al., 2018; Rousseau and Bertolotti, 2018). Thus, the UPS is better suited for the recycling of soluble proteins and not aggregates. In contrast, ALP via macroautophagy (hereafter autophagy) can readily degrade protein aggregates and even damaged cellular organelles (Dikic, 2017). This pathway involves substrate sequestration in double-membranous vesicles called autophagosomes. The autophagosomes then fuse with hydrolytic enzyme-containing lysosomes, thereby generating autolysosomes wherein the cargo is degraded (Pohl and Dikic, 2019).

The proteasome is regulated at multiple levels including transcription, translation, assembly, and posttranslational modifications (Schmidt and Finley, 2014). Our previous studies assigned a central role for the transcription factor Nuclear factor erythroid-derived 2-related factor 1 (Nrf1, also called NFE2L1) in regulating the transcription of proteasome genes (Radhakrishnan et al., 2010, 2014; Vangala and Radhakrishnan, 2019; Vangala et al., 2016). Nrf1 is cotranslationally inserted into the membrane of the endoplasmic reticulum as the precursor p120 and is subject to constant degradation under normal conditions. However, when the cellular proteasome is inhibited, Nrf1 accumulates and is cleaved by the protease DDI2 resulting in p110, an active form of this transcription factor which then migrates to the nucleus and activates proteasome genes (Koizumi et al., 2016; Lehrbach and Ruvkun, 2016). Thus, Nrf1 is designed to sense and respond to proteasome dysfunction, thereby alleviating the resultant proteotoxic stress. Given that proteasome inhibitors such as bortezomib, carfilzomib, and ixazomib are now being used in the clinic as cancer therapeutics, it is important to understand these cellular adaptive responses to proteotoxic stress. These cellular adaptations are also relevant in the context of neurodegenerative diseases characterized by the accumulation of protein aggregates that are known to impair proteasome function (Myeku et al., 2016).

The UPS and ALP have long been considered as two parallel and independent intracellular degradation pathways. However, recent findings have challenged this notion, wherein autophagy has been shown to act as a compensatory pathway when the proteasome is impaired (Bao et al., 2016; Ding et al., 2007; Zhu et al., 2010). Despite this evolving view, the molecular mechanisms that connect these proteolytic pathways are not completely understood. In this study, we present evidence to demonstrate that Nrf1 could act as a molecular link between these two degradation systems and that it could mobilize autophagy when the proteasome is impaired.

## Results

### Nrf1 upregulates the expression of autophagy–lysosomal pathway (ALP) genes in response to proteasome inhibition

**Figure 1.**
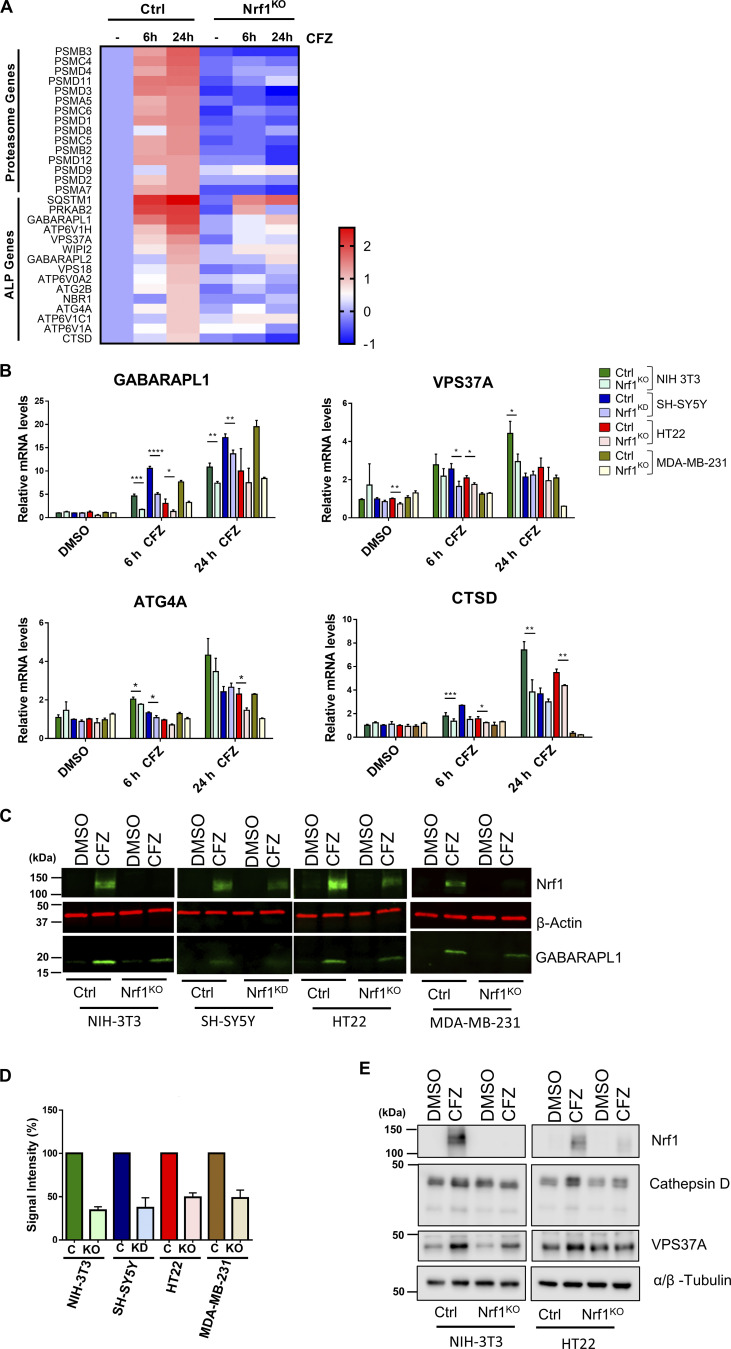
**Nrf1 regulates the expression of ALP genes upon proteasome inhibition. (A)** Heatmap analysis of RNA-seq data (GSE144817) obtained from wild-type (control; ctrl) and Nrf1^KO^ NIH-3T3 cells treated with either DMSO or 200 nM CFZ for 6 or 24 h. Log_2_ fold changes are shown. **(B)** qRT-PCR analysis of NIH-3T3, SH-SY5Y, HT22, and MDA-MB-231 cells that are control (Ctrl) or Nrf1-depleted (KO or KD) were treated with either DMSO or 200 nM CFZ for 6 or 24 h. Expression levels of GABARAPL1, VPS37A, ATG4A, and CTSD were analyzed using gene-specific primers as shown. 18s rRNA or GAPDH levels were used for normalization. **(C)** Western blot analysis of GABARAPL1 protein in NIH-3T3, SH-SY5Y, HT22, and MDA-MB-231 cells treated with either DMSO or 200 nM CFZ for 24 h. β-Actin was used for loading control. **(D)** Quantification of GABARAPL1 signal intensity, normalized to β-Actin signal. **(E)** Western blot analysis of Cathepsin D and VPS37A proteins in control and Nrf1^KO^ NIH-3T3 and HT22 cells treated with either DMSO or 200 nM CFZ for 24 h. α/β-Tubulin was used as the loading control. Three biological replicates for each cell line were used to perform qRT-PCR and Western blotting. P values were calculated by Student’s *t* test. *<0.05, **<0.005, ***<0.0005, ****<0.00005. Source data are available for this figure: SourceData F1.

Our previous studies have demonstrated that inhibition of cellular proteasomes mobilizes the Nrf1 pathway, resulting in de novo synthesis of proteasome genes and subsequent recovery of proteasome activity (Radhakrishnan et al., 2010, 2014; Vangala and Radhakrishnan, 2019; Vangala et al., 2016). To gain further insight into other processes that might also be regulated in an Nrf1-dependent fashion after proteotoxic stress, we analyzed our RNA-sequencing (RNA-seq) dataset with NIH-3T3 wild-type and Nrf1-knockout (Nrf1^KO^) mouse fibroblasts exposed to proteasome inhibitor carfilzomib for either 6 or 24 h (NCBI GEO accession GSE144817 [Vangala et al., 2020]). As expected, we observed a widespread induction of proteasome genes in 6- and 24-h time points in wild-type but not in Nrf1^KO^ samples (Fig. 1 A). Strikingly, we also noticed that several ALP genes were upregulated in an Nrf1-dependent manner, most prominently in the 24-h time point, with some genes also showing a milder induction as early as 6 h (Fig. 1 A). This observation points to the possible existence of an Nrf1-mediated autophagic response as a result of sustained proteotoxic stress.

**Figure S1.**
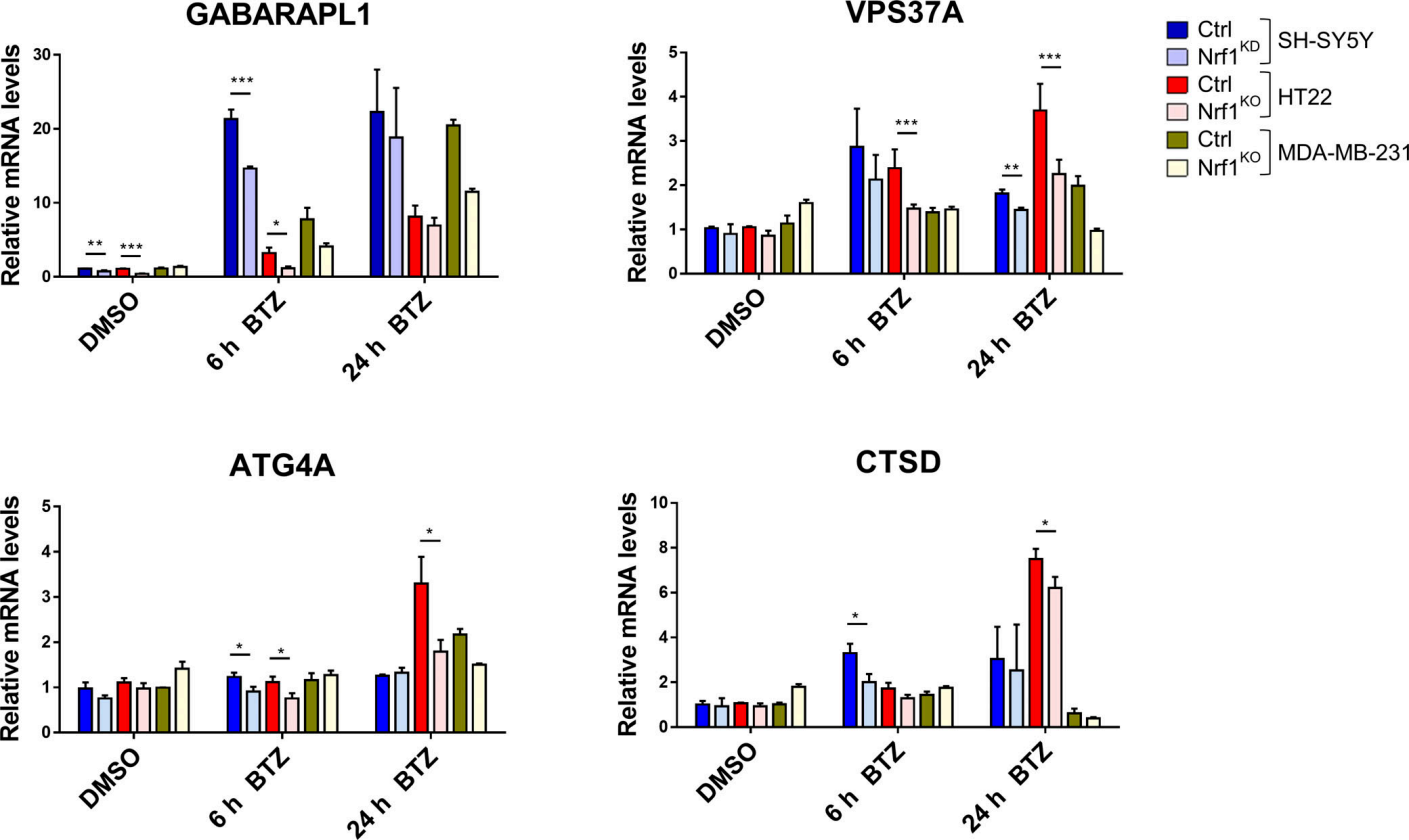
**Nrf1 regulates the expression of ALP genes upon proteasome inhibition.** qRT-PCR analysis of SH-SY5Y, HT22, and MDA-MB-231 cells that are controls (Ctrl) or Nrf1-depleted (KO or KD) were treated with either DMSO or 200 nM bortezomib (BTZ) for 6 or 24 h. Expression levels of GABARAPL1, VPS37A, ATG4A, and CTSD were analyzed using gene specific primers as shown. 18S rRNA or GAPDH levels were used for normalization.

To further evaluate the role of Nrf1 in this response, we used a panel of cell lines of different origin (NIH-3T3 murine fibroblasts, SH-SY5Y human neuroblastoma, HT22 mouse hippocampal neurons, and MDA-MB-231 human breast cancer) that are either control or Nrf1-deficient and assayed for changes in transcript levels of select ALP-related genes in response to treatment with the proteasome inhibitor carfilzomib (CFZ). These genes are involved in various steps of ALP–GABARAPL1 in autophagosome membrane sealing and maturation (Dikic and Elazar, 2018), VPS37A in autophagosome closure (Takahashi et al., 2019), cysteine protease ATG4A in proteolytic activation of GABARAP-related proteins (Dikic and Elazar, 2018), and lysosomal protease CTSD (Cathepsin D) in substrate degradation (Turk et al., 2001). We observed a time-dependent increase in the transcript levels of these genes in control cells in response to CFZ, but this effect was significantly attenuated in Nrf1-deficient cells (Fig. 1 B). To rule out drug-specific effects, we used another proteasome inhibitor, bortezomib (BTZ), and found its effect to be similar to CFZ in robustly activating ALP genes in control when compared with Nrf1-deficient cells (Fig. S1). Consistent with these results, GABARAPL1 protein levels increased in response to CFZ in control cells, but this effect was muted in Nrf1-deficient cells (Fig. 1, C and D). Similarly, Nrf1-dependent increases in protein levels were also observed for Cathepsin D and VPS37A (Fig. 1 E).

**Figure 2.**
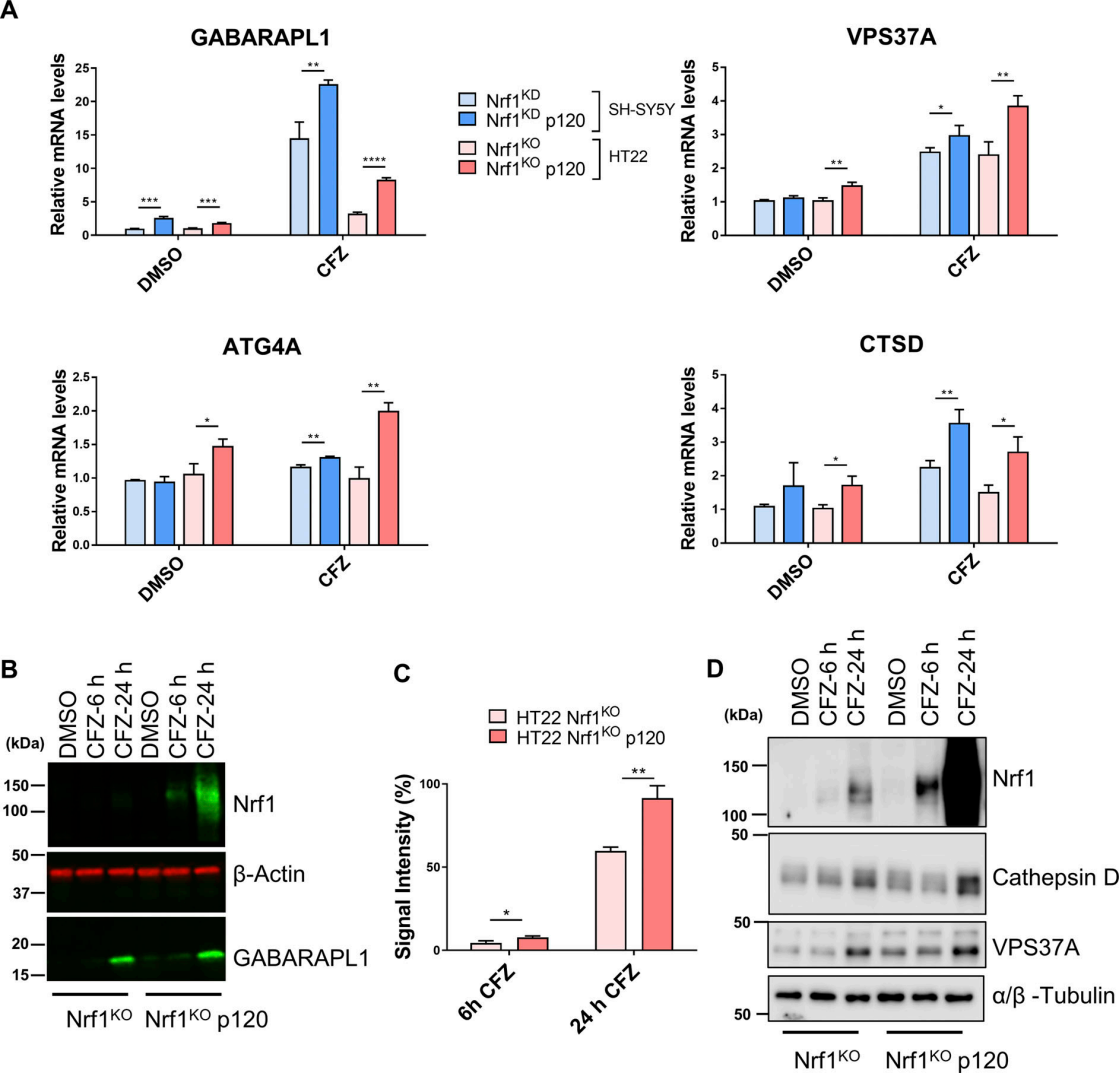
**Adding back Nrf1 in Nrf1-deficient cells rescues suppressed expression of ALP genes upon proteasome inhibition. (A)** SH-SY5Y-Nrf1^KD^ and HT22-Nrf1^KO^ cells were infected with Nrf1(p120). Both SH-SY5Y-Nrf1^KD^, p120 rescue, and HT22-Nrf1^KO^, p120 rescue cells were treated with 200 nM CFZ for 6 h and then analyzed by qRT-PCR to measure the expression levels of indicated genes and mRNA levels of 18s rRNA or GAPDH was used for normalization. **(B)** Western blot analysis of GABARAPL1 and Nrf1 in HT22-Nrf1^KO^ p120 rescue cells treated with either DMSO or 200 nM CFZ for 6 and 24 h. β-Actin was used for loading control. **(C)** Quantification of GABARAPL1 signal intensity, normalized to β-Actin signal. **(D)** Western blot analysis of Cathepsin D and VPS37A in HT22-Nrf1^KO^ and p120 rescue cells treated with either DMSO or 200 nM CFZ for 6 and 24 h. α/β-Tubulin was used as a loading control. Three biological replicates for each cell line were used to perform qRT-PCR and Western blotting. P values were calculated by Student’s *t* test. *<0.05, **<0.005, ***<0.0005, ****<0.00005. Source data are available for this figure: SourceData F2.

**Figure S2.**
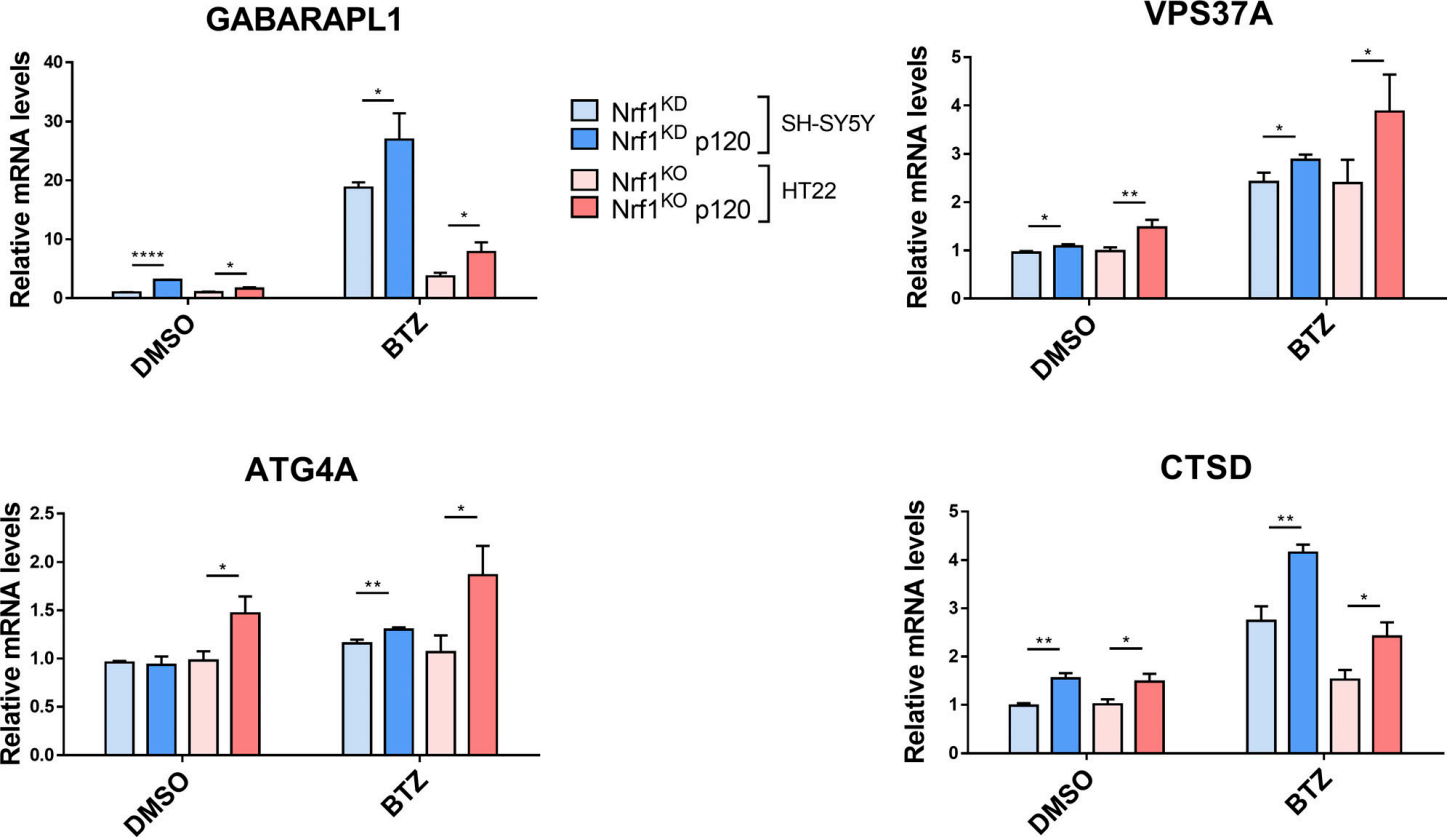
**Adding back Nrf1 in Nrf1-deficient cells rescues suppressed expression of ALP genes upon proteasome inhibition.** SH-SY5Y-Nrf1^KD^ and HT22-Nrf1^KO^ cells were infected with Nrf1(p120). Both SH-SY5Y-Nrf1^KD^, p120 rescue and HT22-Nrf1^KO^, p120 rescue cells were treated with 200 nM BTZ for 6 h and then analyzed by qRT-PCR to measure the expression levels of indicated genes, and mRNA levels of 18S rRNA levels was used for normalization.

Next, we evaluated if reinstating Nrf1 expression in Nrf1-deficient cells could rescue the activation of ALP genes in response to proteasome inhibition. To this end, we overexpressed the Nrf1 precursor p120 in SH-SY5Y and HT22 Nrf1-deficient cells. Regardless of the proteasome inhibitor used (CFZ or BTZ), we saw that the transcripts of ALP genes were upregulated much more in these Nrf1-rescued cell lines when compared with the Nrf1-deficient cell lines (Fig. 2 A and Fig. S2). We observed a similar effect when we examined the protein level of GABARAPL1 (Fig. 2, B and C), Cathepsin D, and VPS37A (Fig. 2 D).

**Figure 3.**
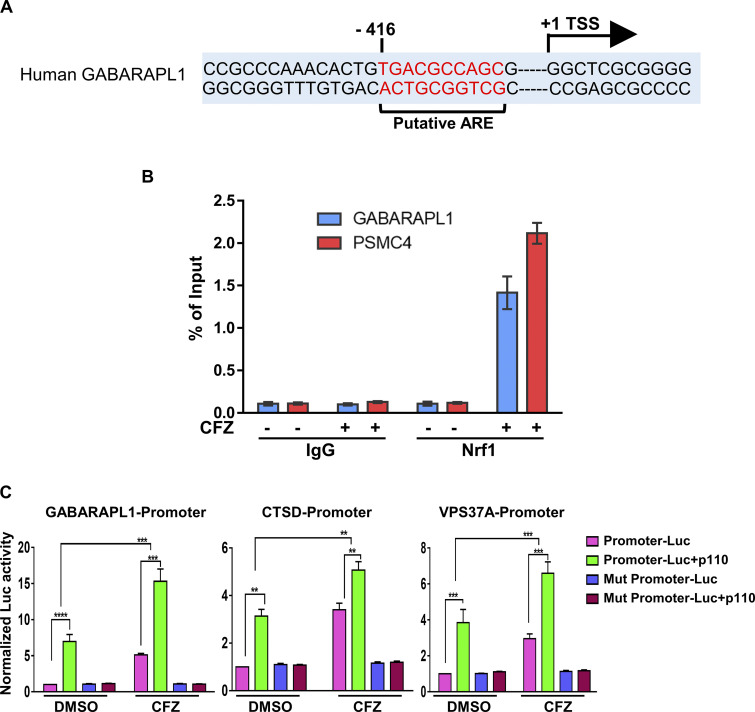
**Nrf1 shows binding to promoter regions of autophagy genes. (A)** Putative ARE sequence close to the transcription start site (+1) of human GABARAPL1. ARE sequence and transcription start site are marked. **(B)** Chromatin immunoprecipitation of GABARAPL1 and PSMC4 (proteasome gene; positive control) in DMSO, CFZ (200 nM/6 h)-treated SH-SY5Y cells were carried out with IgG, Nrf1 antibodies. qPCR analysis was completed with primers flanking the putative ARE sequences (Nrf1 binding) in the promoter region. Nrf1 binding to each gene was expressed as a percentage of the input. Error bars denote mean ± SD (*n* = 3 biological replicates). **(C)** HEK293T cells were transfected with indicated promoter-Luc plasmids, pRL-TK either alone or in combination with Nrf1-p110 for 48 h, followed by CFZ treatment (200 nM; 16 h). Normalized luciferase activities relative to the promoter-luc treated with DMSO are shown. Error bars denote mean ± SD. P values were calculated by two-way ANOVA. *<0.05, **0.005, ***<0.0005, ****<0.00005. (*n* = 3 biological replicates).

To evaluate if Nrf1 could directly transactivate ALP-related genes, we first analyzed the regulatory regions of the human GABARAPL1 gene and found a putative antioxidant response element (ARE; the sequence to which Nrf1 is known to bind [Biswas and Chan, 2010; Wang et al., 2007]) in the proximal promoter region (Fig. 3 A). Importantly, using chromatin immunoprecipitation (ChIP) experiments, we observed recruitment of Nrf1 to this ARE-containing region in response to CFZ in SH-SY5Y cells (Fig. 3 B). To further extend these results, we examined the ChIP-seq datasets in the Encyclopedia of DNA Elements (ENCODE) database (Luo et al., 2020) and found evidence for the binding of Nrf1 to the regulatory regions of multiple ALP-related genes (Table S1).

Furthermore, we tested ∼1 kb promoter regions of GABARAPL1, CTSD, and VPS37A genes, all of which were fused to firefly luciferase. We found that these promoters were induced in response to overexpression of Nrf1 p110 (active form) and/or treatment with CFZ, but this effect was abolished when putative ARE sites were mutated (Fig. 3 C). Together, these findings are consistent with a model in which proteotoxic stress-induced Nrf1 transactivates ALP genes via direct binding to their regulatory regions.

### Nrf1 is necessary to induce autophagic flux in response to proteasome inhibition

**Figure 4.**
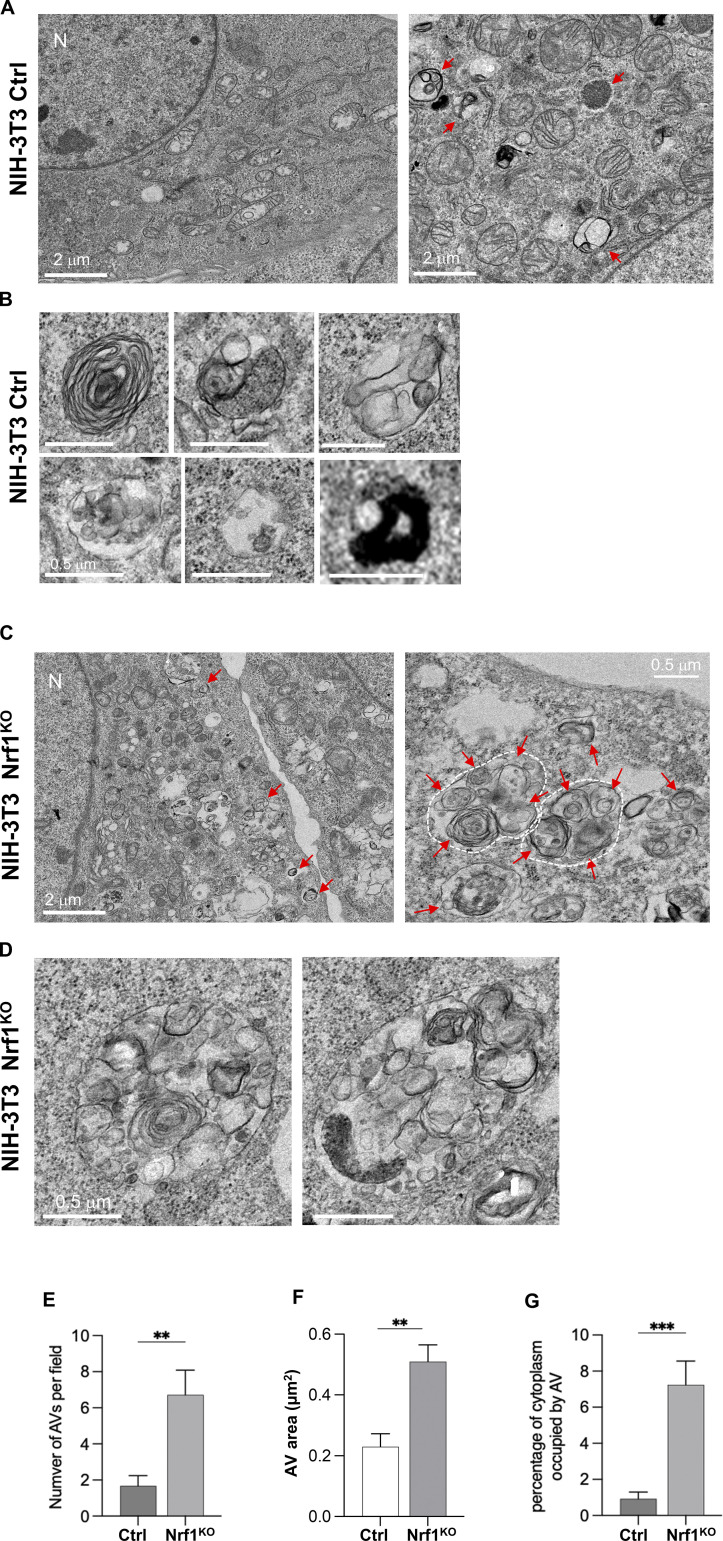
**Nrf1**^**KO**^ **cells display defective autophagy in response to proteasome inhibition. (A and B)** Transmission electron microscopy (TEM) images of NIH-3T3 control (ctrl) cells treated with 200 nM CFZ for 20 h with B showing zoom insets. **(C and D)** TEM images of Nrf1^KO^ cells treated with 200 nM CFZ for 20 h with D showing zoom insets. Multiautophagosomal structures (white dash outlines) are visible in the Nrf1^KO^ cells. N: nucleus; red arrows: autophagic vacuoles (AV). **(E–G)** Quantification shows the number of AV per field in E, the area of AV in F, and the percentage of cytoplasm occupied by AV in G. P values were calculated by Student’s *t* test. **P < 0.01, ***P < 0.001, *n* = 9–10 fields.

On a functional level, to see if ablation of Nrf1 results in a difference in the extent of autophagy, we first visualized NIH-3T3 cells treated with CFZ by transmission electron microscopy (TEM). We found that relative to the control, Nrf1-deficient cells accumulated multiautophagosomal structures with cytoplasmic components inside them (Fig. 4, A–D). Interestingly, a double membrane was visible in these structures, suggestive of reduced autophagic flux. Also, the total number and area of autophagic vacuoles (AVs) and the percentage of cytoplasm occupied by AVs were all increased in CFZ-treated Nrf1^KO^ cells (Fig. 4, E–G). These observations from the TEM images imply a marked defect in the autophagic process, thus underscoring the importance of Nrf1 in this context.

**Figure 5.**
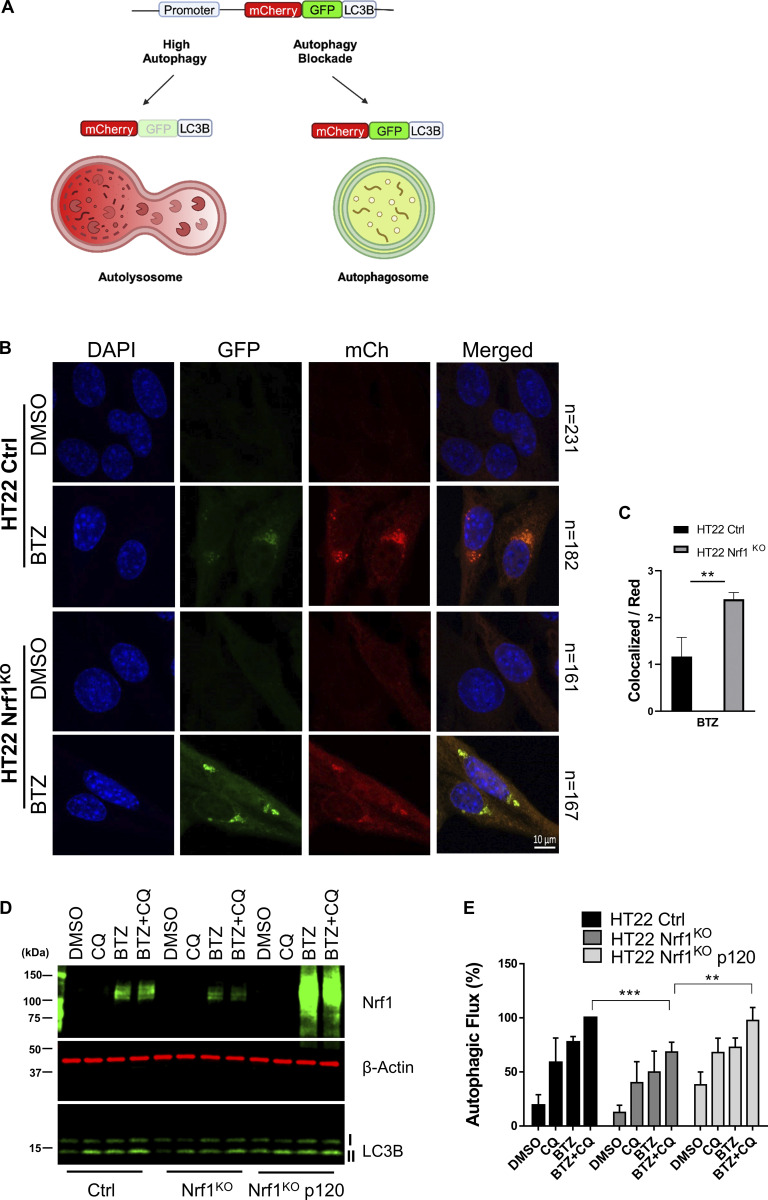
**Nrf1 can induce autophagy upon proteasome inhibition. (A)** Schematic representation of the mCherry-GFP-LC3B fluorescence reporters. **(B)** Representative confocal microscopy images of HT22 wild-type (control; ctrl) and Nrf1^KO^ cells, stably expressing mCh-GFP-LC3B construct for detecting autophagic flux. Cells were treated either with DMSO or 200 nM BTZ for 20 h. The scale bar represents 10 μm. **(C)** The ratio of colocalized puncta signal to red puncta signal and normalizing this value to total cell number in each condition is shown. Number of cells analyzed in each sample is noted next to the images in B. **(D)** Western blot analysis of Nrf1 and LC3B in HT22-ctrl, Nrf1^KO^, Nrf1^KO^ p120 cells after treatment with either DMSO, 200 nM BTZ (20 h), 60 μM CQ (3 h), or both BTZ (20 h) and CQ (3 h). β-Actin was used as a loading control. **(E)** Percent autophagic flux determined by normalizing LC3B-II levels to β-Actin levels from E. Three biological replicates for both microscopy and Western blotting were used, and P values were calculated by Student’s *t* test. **<0.05, **<0.005, ***<0.0005. Source data are available for this figure: SourceData F5.

To further delineate the role of Nrf1 in inducing compensatory autophagy, we decided to examine autophagic flux in HT22 cells. LC3 protein is essential for the execution of autophagy and is widely used as a marker to assess autophagy activation (Klionsky et al., 2016). Also, our RNA-seq dataset described earlier (Vangala et al., 2020 and Fig. 1 A) did not show a significant difference for LC3 at the transcript level in response to CFZ and/or Nrf1 status, making this a reliable marker in this context. We first used a tandem monomeric mCherry-GFP-tagged LC3B protein (N’Diaye et al., 2009) to monitor autophagic flux (Fig. 5 A). GFP is quenched under acidic conditions, while mCherry is stable. Therefore, colocalization of mCherry and GFP signals indicates autophagosomes, whereas mCherry signal alone shows autolysosomes due to quenching of GFP in lysosomes. In the case of autophagy induction, this reporter ends up primarily localized to the lysosome thus yielding a significant amount of red puncta; however, lower or blockade in autophagosome maturation causes an increase in colocalized greenish yellow puncta and a decrease in red puncta. With the help of this reporter, we observed that BTZ-induced autophagic flux was attenuated in HT22 Nrf1^KO^ cells when compared with wild-type control (Fig. 5 B). Next, from three independent experiments, by dividing colocalized puncta area (greenish yellow) with the red puncta area and by normalizing this value to the total cell number, we calculated the ratio of autophagosomes to autolysosomes. In BTZ-treated samples, this ratio was higher in Nrf1^KO^, thus confirming the attenuation of autophagic flux in these cells when compared with the control (Fig. 5 C).

**Figure S3.**
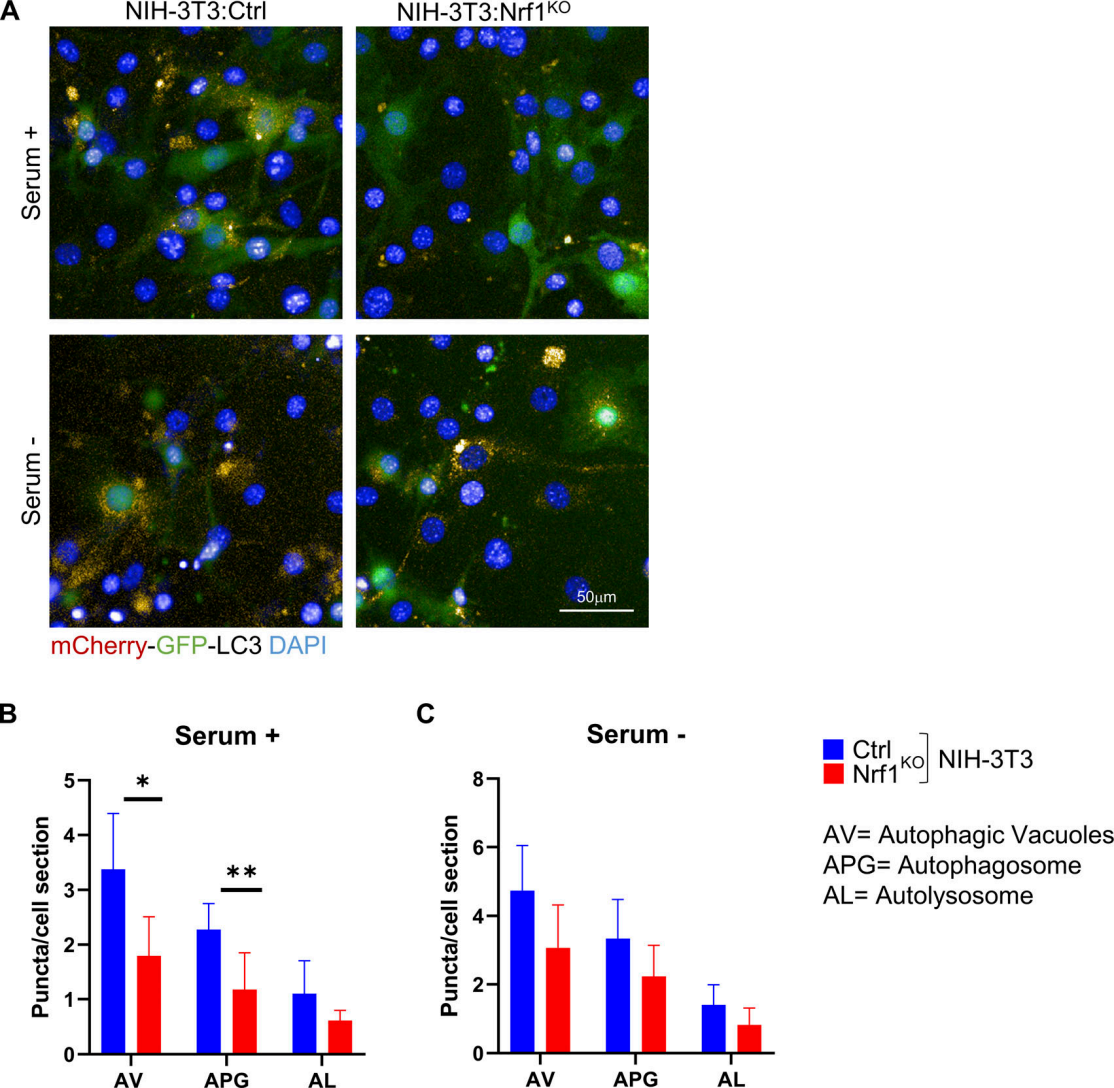
**Nrf1**^**KO**^ **cells display defects in basal and starvation-induced autophagy.** NIH-3T3 cells that are wild-type control (ctrl) or Nrf1^KO^ expressing the tandem reporter mcherry-GFP-LC3B were incubated in serum-supplemented (serum +) or serum-deprived (serum −) media for 6 h. The number of red puncta (autophagic vacuoles; AV), yellow puncta (autophagosomes; APG), and red only puncta (autolysosomes; AL) were quantified as the average number of fluorescent puncta per cell. **(A–C)** Representative images are shown in A, and the quantification of puncta is shown in B and C. Scale bar represents 50 μm. Differences were significant for *P < 0.05 and **P < 0.001.

Next, we wanted to validate our observations via an orthogonal setup. Autophagic flux can be tested by using an autophagy inhibitor with or without the potential autophagy inducer and then examining LC3-II turnover by Western blot (Klionsky et al., 2016). Specifically, pro-LC3 protein was first cleaved by ATG4 to form LC3-I, which was then lipidated to generate LC3-II. This lipidated LC3-II then conjugates to autophagosomes and recruits autophagic cargo by interacting with autophagy receptors such as p62 (Melia et al., 2020). Interestingly, both inhibition and activation of autophagy can result in elevated LC3-II levels due to inefficient degradation of LC3-II and increased production of LC3-II, respectively (Mizushima and Murphy, 2020). Accordingly, we used a lysosomal inhibitor, chloroquine (CQ) to inhibit LC3 degradation and BTZ to induce autophagy. Wild-type HT22 cells showed an increase in LC3-II level when treated with either CQ or BTZ compared with DMSO-only treatment (Fig. 5 D). Moreover, when we treated wild-type HT22 cells with both CQ and BTZ, we observed an even higher increase in LC3-II levels, compared with single treatments, confirming the role of BTZ in inducing autophagic flux (autophagosome formation and clearance; Fig. 5 D). In contrast, we observed that LC3-II protein levels were significantly less in Nrf1^KO^ cells compared with control cells in response to both CQ and BTZ (Fig. 5 E), suggesting an impairment of autophagic flux in Nrf1^KO^ cells. Next, we examined whether restoring Nrf1 levels in Nrf1^KO^ cells can rescue the autophagic flux impairment. We observed that overexpression of precursor Nrf1 p120 in Nrf1^KO^ cells significantly increased LC3-II levels, compared to Nrf1^KO^ cells, when treated with both CQ and BTZ (Fig. 5, D and E). Overall, our data suggest that Nrf1 is required for activation of compensatory autophagy upon proteasome inhibition. Consistent with this theme, we also found that NIH-3T3 Nrf1^KO^ cells display defects in basal and starvation-induced autophagy, manifested as an overall reduction in number of autophagic compartments in both conditions when compared with control cells (Fig. S3).

### Nrf1 is required to clear aggresomes associated with proteasome inhibition

**Figure 6.**
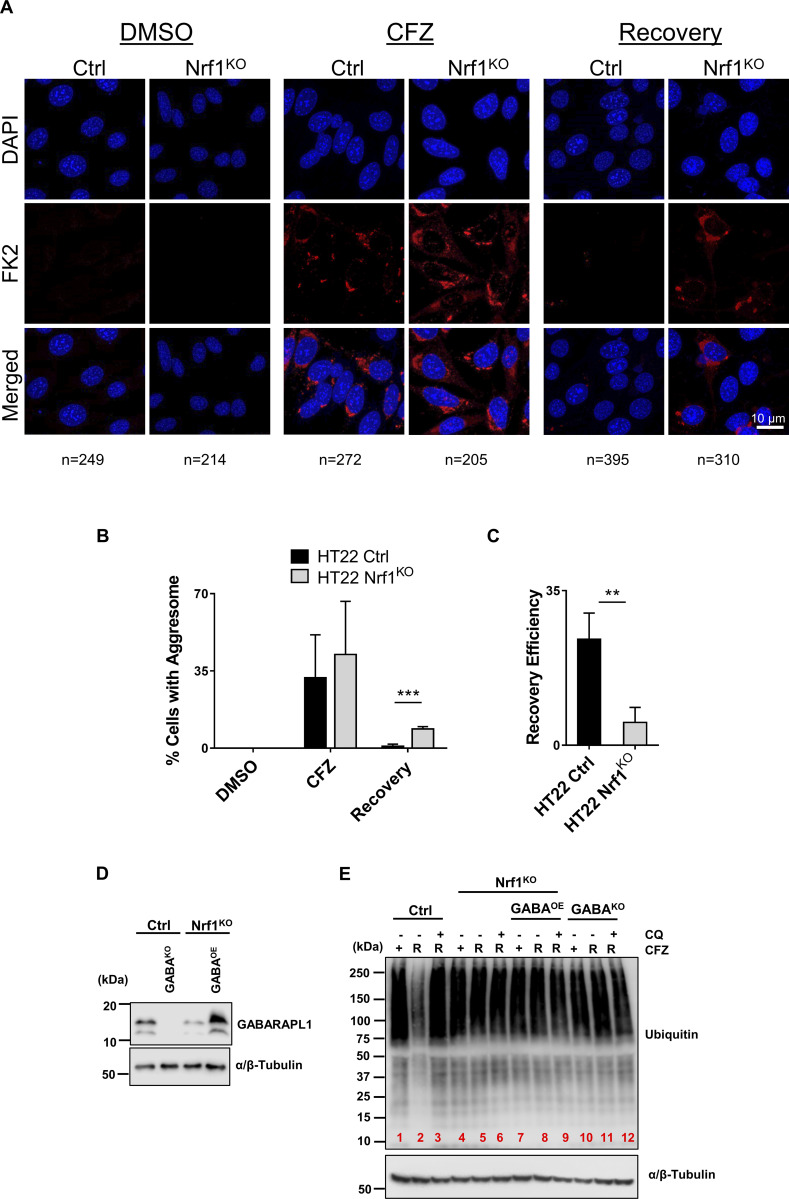
**Nrf1 is required to clear aggresomes that are triggered by proteasome inhibition. (A)** HT22 wild-type (control; ctrl) and Nrf1^KO^ cells were treated with 50 nM CFZ for 20 h, then were washed and incubated with fresh media for another 20 h (Recovery period). Aggresomes were detected by confocal microscopy using FK2 stain. Scale bar represents 10 μm. **(B)** Percentage of cells with aggresomes under each condition (DMSO, CFZ and recovery) for both ctrl and Nrf1^KO^ cells are plotted, based on confocal microscopy analysis. The number of cells analyzed in each sample is noted underneath the images in A. **(C)** Recovery efficiency of ctrl and Nrf1^KO^ cells was calculated by dividing CFZ values by recovery values of ctrl and Nrf1^KO^ cells in B. **(D)** Western blot analysis of GABARAPL1 overexpression in Nrf1^KO^ cells. α/β-Tubulin was used as the loading control. **(E)** HT22 control (EV), GABA^KO^ (GABARAPL1 knockout), Nrf1^KO^, Nrf1^KO^ GABA^OE^ (GABARAPL1 overexpression) cells were treated with 50 nM CFZ for 20 h, then washed, and either treated with 60 μM CQ or fresh complete media for a 20 h recovery (R). The samples were analyzed by immunoblotting with anti-ubiquitin antibody. α/β-Tubulin was used as the loading control. Three biological replicates were used for confocal microscopy analysis. P values were calculated by Student’s *t* test. **<0.05, ***<0.005. Source data are available for this figure: SourceData F6.

Functional decline in the activity of the proteasomes through aging, genetic mutations, or environmental stress can lead to the aggregation of misfolded and unwanted proteins (Lopez-Otin et al., 2013). These toxic aggregates such as in the case of neurological diseases are also known to inhibit proteasomes (Myeku et al., 2016). These aggregates form perinuclear inclusion bodies that are called aggresomes and can be cleared by a selective type of autophagy termed aggrephagy (Bauer et al., 2023). To test whether Nrf1 plays a role in the aggresome clearance pathway, we treated HT22 wild-type and Nrf1^KO^ cells with CFZ and observed them under the confocal microscope. Treatment of these cells with CFZ induced aggresome formation, which was evident by staining these cells with a polyubiquitin antibody FK2 (Fig. 6 A). We then removed CFZ and let the cells recover in fresh complete media and observed for residual aggresomes. While wild-type cells were able to clear aggresomes almost completely, this effect was significantly impaired in Nrf1^KO^ cells (Fig. 6, A–C).

Given the prominent induction of GABARAPL1 in response to proteasome inhibition, we next asked if its reconstitution alone in Nrf1^KO^ cells could rescue the defect in aggrephagy. To this end, we overexpressed GABARAPL1 in Nrf1^KO^ HT22 cells and in parallel generated HT22 GABARAPL1^KO^ cells as a control (Fig. 6 D). Using these cell lines, we optimized a biochemical version of the aggresome clearance assay, where CFZ-induced ubiquitin-positive aggregates in detergent-insoluble fractions were visualized using anti-ubiquitin antibodies in an immunoblot format. In control cells, CFZ-induced aggregates were efficiently cleared after CFZ was washed out (Fig. 6 E, lanes 1 versus 2), and this clearance was completely blocked when chloroquine was added to the recovery medium (Fig. 6 E, lane 3), clearly establishing this process as ALP-dependent. In contrast, Nrf1^KO^ cells were unable to clear the aggregates (Fig. 6 E, lanes 4 versus 5), and reconstitution of GABARAPL1 was insufficient to rescue this defect (Fig. 6 E, lanes 7 versus 8). The control GABARAPL1^KO^ cells were unable to clear the aggregates (Fig. 6 E, lanes 10 versus 11), as expected, because of the critical role of GABARAPL1 in ALP (Dikic and Elazar, 2018).

**Figure S4.**
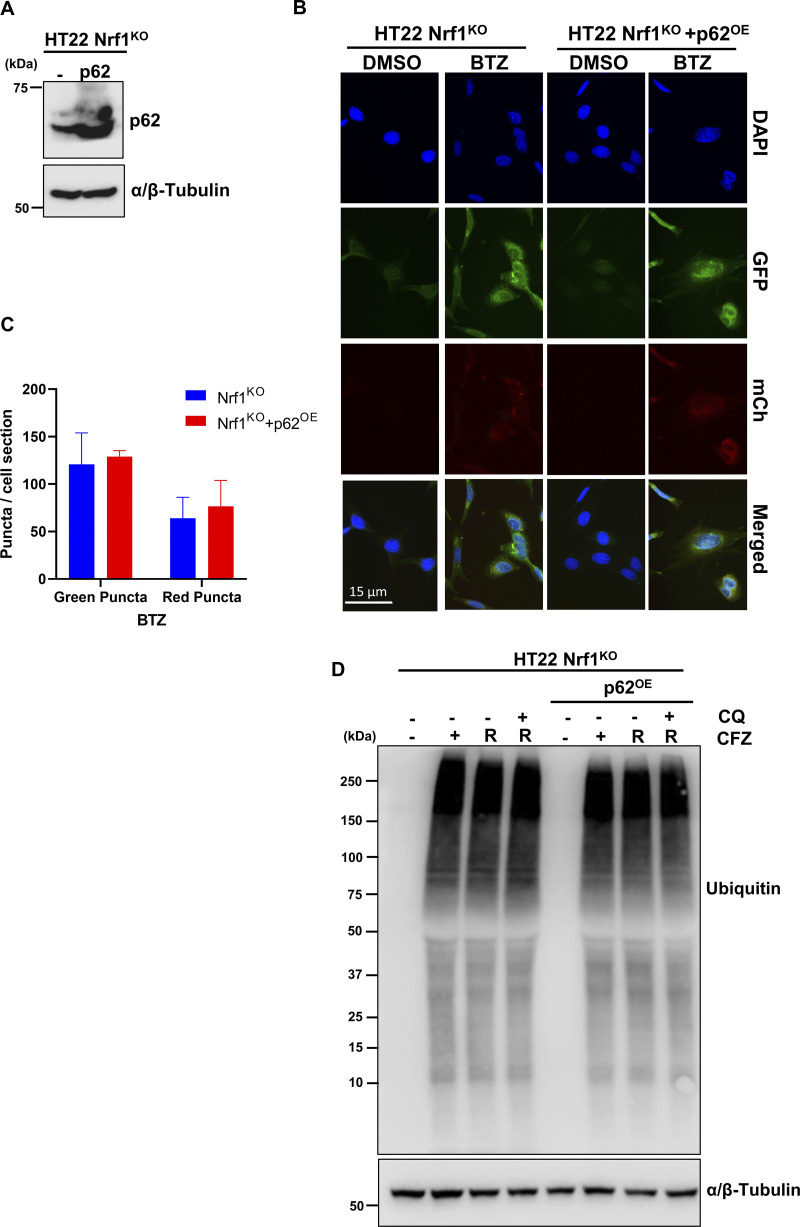
**p62 over expression does not rescue aggresome clearance in Nrf1**^**KO**^ **cells. (A)** Confirmation Western blot for p62 protein expression in HT22 Nrf1^KO^ and HT22 Nrf1^KO^ +p62^OE^ overexpression cells. α/β-Tubulin was used for loading control. **(B)** Representative images for autophagic flux in HT22 mCherry-GFP-LC3B Nrf1^KO^ and mCherry-GFP-LC3B Nrf1^KO^ +p62^OE^ cells treated for 20 h with DMSO or 200 nM BTZ. Images taken at 63×. Scale bar represents 15 μm. **(C)** Quantification of red and green puncta from BTZ treatment in panel B, signal is normalized to total cell number for each condition (Nrf1^KO^ *n* = 52 fields, Nrf1^KO^ + p62^OE^ *n* = 45 fields). **(D)** HT22 Nrf1^KO^ and Nrf1^KO^+ p62^OE^ cells were treated with 50 nM CFZ for 20 h, then washed and either treated with 60 μM CQ or fresh complete media for a 20 h recovery (R). Western blot analysis of Ubiquitin after treatment and recovery, α/β-Tubulin was used for loading control. (*n* = 3 biological replicates). Error bars denote mean ± SD. Source data are available for this figure: SourceData FS4.

Next, based on a previous report that p62/SQSTM1, an autophagy adaptor, is induced in response to proteasome inhibition (Sha et al., 2018), we asked if overexpression of this gene alone could rescue the ALP-related defects in Nrf1^KO^ cells. However, overexpression of p62 (Fig. S4 A) neither rescued the defect in compensatory autophagic flux (Fig. S4, B and C) nor the aggregate/aggresome clearance (Fig. S4 D) in Nrf1^KO^ cells. Taken together, our results suggested that Nrf1 might need to induce a battery of ALP-related genes to clear aggresomes efficiently after proteasome inhibition.

### Nrf1 is sufficient to induce ALP

**Figure 7.**
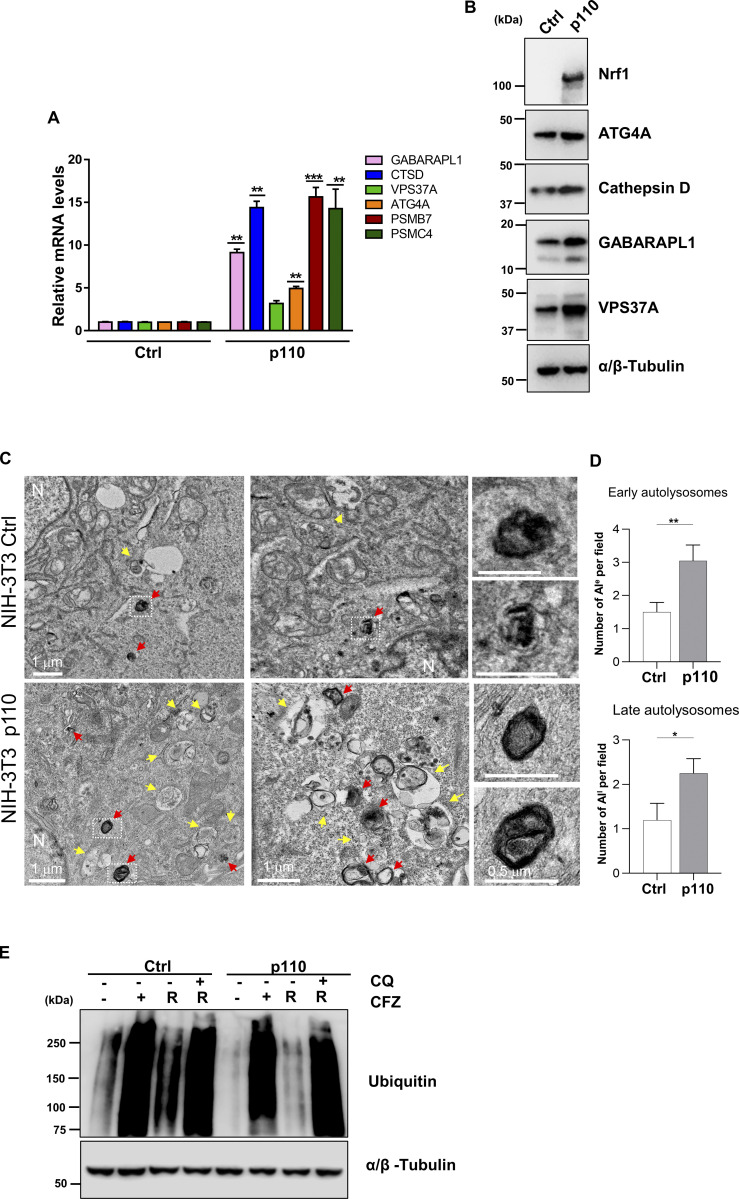
**Nrf1 is sufficient for induction of autophagy. (A)** qRT-PCR analysis of GABARAPL1, CTSD, VPS37A, ATG4A, PSMB7, and PSMC4 in NIH-3T3 control (empty vector; EV) and p110 overexpressing cells. 18s rRNA level was used for normalization. **(B)** Western blot analysis of Nrf1, ATG4A, Cathepsin D, GABARAPL1, and VPS37A in NIH-3T3 control and p110 overexpressing cells. α/β-Tubulin was used as the loading control. **(C)** TEM images of NIH-3T3 control (Ctrl) and p110 overexpressing cells. N: nucleus. Yellow arrows: early autolysosomes (AL^e^). Red arrows: late autolysosomes (AL^l^). Zoom inset shows late autolysosomes. **(D)** Quantification shows the number of AL^e^ and AL^l^ per field. **(E)** NIH-3T3 control (EV) and p110 cells were treated with 50 nM CFZ for 20 h then washed and either treated with 60 μM CQ or fresh complete media for a 20 h recovery (R). Western blot analysis of Ubiquitin after treatment and recovery, α/β-Tubulin was used for loading control. (*n* = 3 biological replicates). Error bars denote mean ± SD. P values for qRT-PCR were calculated by two-way ANOVA, *<0.05, **0.005, ***<0.0005. P values for TEM were calculated by Student’s *t* test. *P < 0.05, **P < 0.01, *n* = 20 fields. Source data are available for this figure: SourceData F7.

Our results so far demonstrate that Nrf1 is required for the induction of compensatory autophagy and aggresome clearance in response to proteasome inhibition. We next asked if increasing Nrf1 levels could by itself mobilize ALP in the absence of other signals provided by proteasome inhibition. To this end, we overexpressed p110, the proteolytically processed and active form of Nrf1 in NIH-3T3 cells. We observed that, in the absence of proteasome inhibitor treatment, overexpression of p110 was able to induce the transcripts and protein levels of representative ALP genes GABARAPL1, CTSD, VPS37A, and ATG4A to varying levels (Fig. 7, A and B). As a control, we measured proteasome genes PSMB7 and PSMC4 mRNA levels, which were induced as expected in response to p110 (Fig. 7 A).

Next, we compared TEM images of cells overexpressing p110 with that of control cells and found that p110 overexpression resulted in a significant increase in the levels of both early and late autolysosomes (Fig. 7, C and D). This suggests that p110-overexpressing cells could be endowed with an increased ability to stimulate autophagic flux. To test this hypothesis, we then assessed the ability of p110-overexpressing cells to clear aggregates caused by proteasome inhibition. Using the anti-ubiquitin immunoblot assay, we observed that control cells were able to clear CFZ-induced aggregates in a manner dependent on functional autophagy since this clearance was blocked by the addition of chloroquine (Fig. 7 E). Interestingly, this clearance was dramatically enhanced in p110-overexpressing cells, confirming the notion that p110 alone could mobilize ALP to a significant level (Fig. 7 E).

### Deficiency of NGLY1, a positive upstream regulator of Nrf1, causes attenuation of compensatory autophagy and aggrephagy

**Figure 8.**
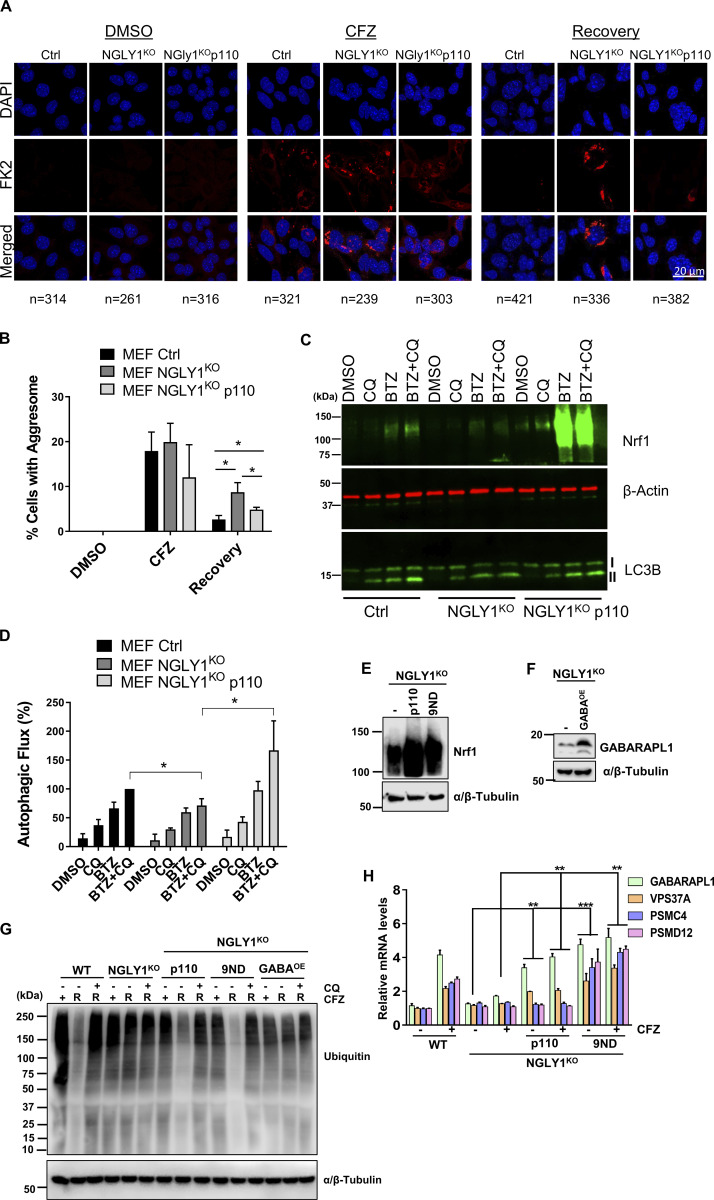
**Deficiency of NGLY1 causes inhibition of compensatory autophagy and aggrephagy, which can be rescued by transcriptionally active Nrf1. (A)** Confocal images of FK2 labeled MEF ctrl, NGLY1^KO^, and NGLY1^KO^ p110 cells after treating them with DMSO or 25 nM CFZ for 20 h. Recovery samples were washed out after CFZ treatment and incubated in fresh complete media for 20 h. Scale bar represents 20 μm. **(B)** Analysis of the percentage of cells with aggresomes under each condition from (5A) are plotted, based on the confocal microscopy analysis. Number of cells analyzed in each sample is noted underneath the images in B. **(C)** Western blot analysis of LC3B, and Nrf1 in MEF-ctrl, NGLY1^KO^, and NGLY1^KO^ cells expressing p110 construct treated with DMSO, 200 nM BTZ (20 h), 60 μM CQ (3 h) or both BTZ (20 h) and CQ (3 h). β-Actin was used as a loading control. **(D)** Percent autophagic flux after normalizing LC3B-II levels to β-Actin levels from 8C. **(E)** Confirmation Western blot for Nrf1 protein expression in MEF NGLY1^KO^, NGLY1^KO^ p110 overexpression, and NGLY1^KO^ 9ND overexpression cells treated with CFZ (200 nM/4 h). α/β-Tubulin was used as the loading control. **(F)** Confirmation Western blot for GABARAPL1 protein expression in MEF NGLY1^KO^, and NGLY1^KO^ GABARAPL1 over expression cells (GABA^OE^) cells. α/β-Tubulin was used for loading control. **(G)** MEF control (WT), NGLY1^KO^, NGLY1^KO^ p110, and NGLY1^KO^ 9ND cells were treated with 50 nM CFZ for 20 h, then washed and either treated with 60 μM CQ or fresh complete media for a 20 h recovery (R). Western blot analysis of ubiquitin after treatment and recovery, α/β-Tubulin was used for loading control. **(H)** MEF control (WT), NGLY1^KO^, NGLY1^KO^ p110, and NGLY1^KO^ p110-9ND cells were treated with 200 nM CFZ for 20 h. qRT-PCR analysis of ATG4A, GABARAPL1, CTSD, VPS37A, PSMD12, and PSMC4, mRNA levels were normalized to 18s rRNA. (*n* = 3 biological replicates). Error bars denote mean ± SD. Three biological replicates for qRT-PCR, microscopy and Western blotting were used. P values were calculated by either Student’s *t* test or two-way ANOVA. *<0.05, **<0.005, ***<0.0005 Source data are available for this figure: SourceData F8.

N-linked glycosylation is a posttranslational modification of Asparagine residues of proteins that are in the ER lumen (Suzuki and Fujihira, 2024; Suzuki et al., 2016; Suzuki and Yoshida, 2022). NGlycanase-1 (NGLY1) is an evolutionarily conserved enzyme that removes N-linked glycosylation from cytosolic proteins (de-glycosylation). NGLY1 deficiency causes a rare congenital disorder characterized by developmental delay, seizures, and neurological and liver malfunction among many other symptoms (Enns et al., 2014). On the molecular level, we and others have demonstrated that in this disease, deficiency of NGLY1 results in defective Nrf1 maturation due to the lack of its deglycosylation (Lehrbach et al., 2019; Lehrbach and Ruvkun, 2016; Tomlin et al., 2017). Given that deglycosylation of Nrf1 is critical for its transcriptional activity, we asked if NGLY1 deficiency causes impairment of compensatory autophagy and aggrephagy in response to proteotoxic stress. To this end, we used mouse embryonic fibroblast (MEF) cells that are wild-type, NGLY1^KO^, and NGLY1^KO^ stably expressing p110, the activated form of Nrf1 that does not require NGLY1 activity (NGLY1^KO^ p110). First, to test if clearance of aggresomes is impaired in NGLY1^KO^ cells, we treated wild-type, NGLY1^KO^, and NGLY1^KO^ p110 MEF cells with CFZ and let them recover in fresh complete media. Then, we stained the aggresomes with FK2 antibody and imaged them under the confocal microscope. As expected, all MEF cells induced aggresomes after proteasome inhibition (Fig. 8 A); however, wild-type cells cleared the aggresomes more efficiently than the NGLY1^KO^ cells (Fig. 8, A and B). Importantly, the addition of Nrf1 p110 in NGLY1^KO^ cells resulted in the rescue of impaired aggresome clearance phenotype associated with NGLY1 deficiency (Fig. 8, A and B), suggesting the requirement of Nrf1 in this process.

Next, we tested the autophagic flux in this disease model by Western blotting for LC3B-II. Treatment of wild-type MEF cells with CQ and BTZ resulted in elevated LC3B-II levels compared with DMSO (Fig. 8 C). As expected, treating cells with both CQ and BTZ caused an even higher LC3B-II level in wild-type cells, confirming that autophagic flux is activated in these cells by BTZ treatment. However, cotreatment of CQ and BTZ did not result in an increase in LC3B-II levels in NGLY1^KO^ cells (Fig. 8, C and D) compared with single treatments, suggesting that autophagic flux was impaired in NGLY1^KO^ cells. Importantly, expression of Nrf1 p110 in NGLY1^KO^ cells was able to rescue this impairment and restore autophagic flux, which was evident by elevated LC3B-II levels in response to CQ+BTZ combination treatment (Fig. 8, C and D).

Elegant studies in worms and mammalian cells, primarily using NGLY1 model systems, have demonstrated that NGLY1-mediated deglycosylation of Nrf1 serves a surprising sequence editing function by converting certain N-glycosylated asparagine residues to aspartic acid (Lehrbach, 2022; Lehrbach et al., 2019; Ruvkun and Lehrbach, 2023; Yoshida et al., 2021). This sequence editing was found to be critical for maintaining the optimal activity of Nrf1 in the induction of proteasome genes. To check the importance of this phenomenon in proteasome inhibitor-mediated ALP induction, we compared the effect of p110 wild-type with that of the sequence edited version p110 9ND (where nine residues are changed to Asp) in NGLY1^KO^ MEF cells (Fig. 8 E). As a control, we also used overexpression of GABARAPL1 in these NGLY1^KO^ MEF cells (Fig. 8 F). First, using the anti-ubiquitin immunoblot assay, we observed that in NGLY1^KO^ MEFs, wild-type p110 was able to clear CFZ-induced aggregates to the level of wild-type MEFs (Fig. 8 G). Interestingly, p110 9ND was able to elicit aggregate clearance markedly better than wild-type p110 in NGLY1^KO^ MEFs (Fig. 8 G). On the other hand, overexpression of GABARAPL1 alone in this context was unable to help clear the aggregates (Fig. 8 G).

To further contrast the difference between wild-type p110 and the 9ND version in NGLY1^KO^ cells, we measured the mRNA levels of representative proteasome (PSMC4 and PSMD12) and ALP genes (GABARAPL1 and VPS37A). Proteasome inhibitor CFZ was able to induce all of these genes in wild-type but not in NGLY1^KO^ MEFs as expected (Fig. 8 H). Interestingly, we found that wild-type p110 could induce ALP genes, but not proteasome genes (Fig. 8 H). In contrast, p110 9ND was able to induce proteasome genes and also activate ALP genes modestly better than wild-type (Fig. 8 H). Together, our results imply a differential requirement for Nrf1 sequence editing among its target genes in these MEF cells. Of note, as described earlier, wild-type p110 was able to induce proteasome genes in NIH-3T3 cells (Fig. 7 A), which was different from what we observed here in MEF cells, and this could be due to cell type-specific differences. Overall, our data point to a critical role for Nrf1 in mediating proteasome inhibitor-induced compensatory autophagy and aggrephagy in this MEF NGLY1 disease model.

## Discussion

**Figure 9.**
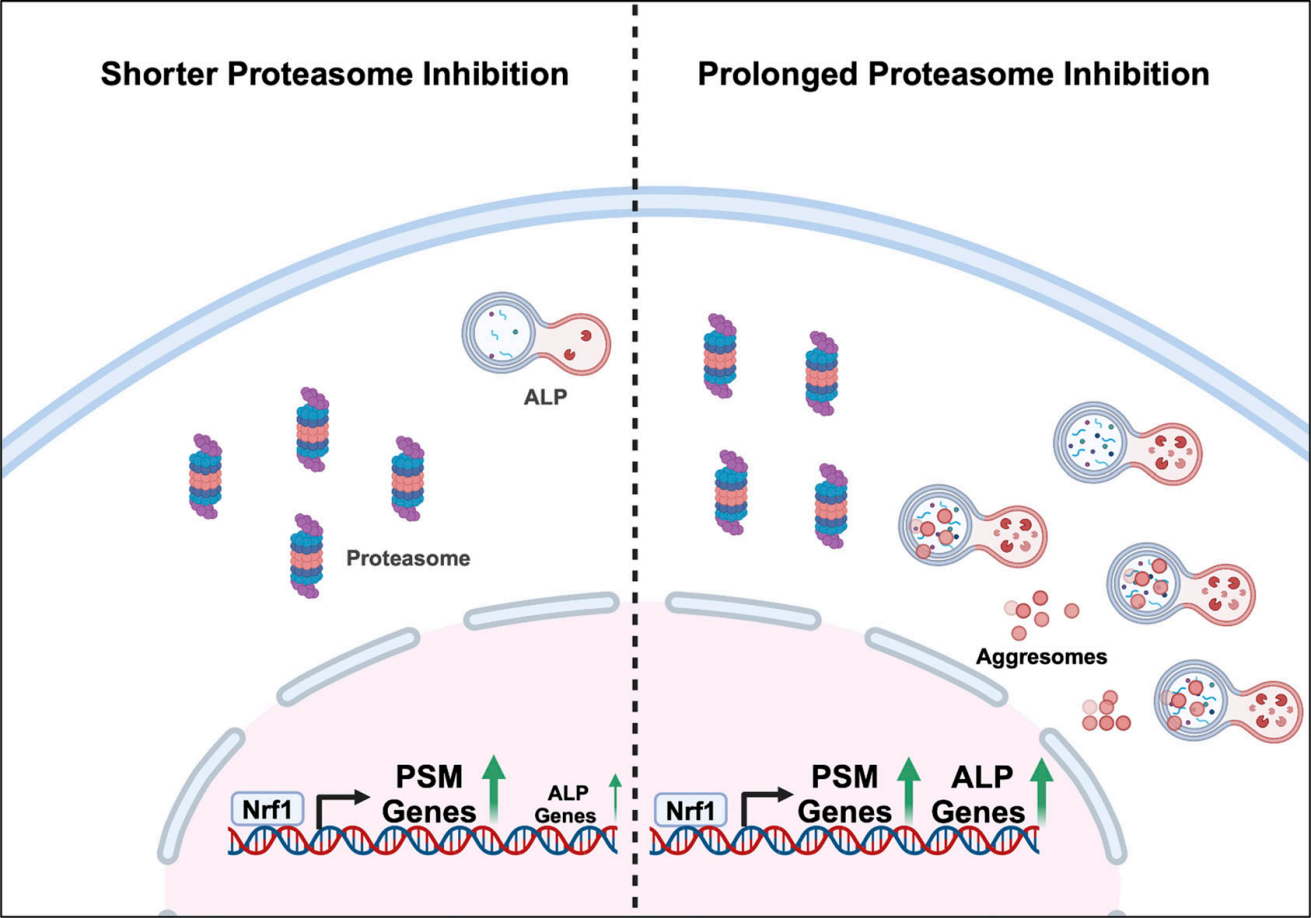
**Dual regulation of proteasome and ALP by Nrf1.** Protein degradation pathways, the ubiquitin proteasome system (UPS), and the autophagy–lysosomal pathway (ALP), are essential for maintaining proteostasis in the cell. During proteotoxic stress or when the proteasome is inhibited, Nrf1 upregulates both protein degradation pathways in a time-dependent manner. With shorter proteasome inhibition, shown on the left, proteasome (PSM) genes are robustly induced. However, after prolonged proteasome inhibition, shown on the right, there is an increase in aggresomes, which are not cleared through the UPS. This longer inhibition results in an increase in upregulation of multiple ALP genes, alongside the PSM genes, to clear the aggresomes. This allows the cell to maintain proteostasis and promote cell survival.

Our earlier work firmly established a role for the transcription factor Nrf1 in responding to proteotoxic stress caused by the inhibition of proteasome activity (Radhakrishnan et al., 2010, 2014; Vangala and Radhakrishnan, 2019; Vangala et al., 2016). This response is characterized by transcriptional upregulation of proteasome genes resulting in the bounce-back of proteasome activity (Steffen et al., 2010). Our current study expands the role of Nrf1 in this context as a transcriptional regulator of ALP genes in addition to the proteasome genes. Our RNA-seq analysis indicates that while proteasome genes are robustly induced early on, many of the ALP genes are induced more strongly at a later time point. By increasing autophagic flux in response to prolonged proteasome inhibition, Nrf1 provides the cells with an additional route to cope with proteotoxic stress. Proteasome inhibitors are known to trigger the formation of ubiquitylated protein aggregates (Kopito, 2000). Given that these aggregates are poor substrates of the proteasome, the ability of Nrf1 to induce autophagy could provide the cells with the capacity to degrade these aggregates via aggrephagy, as shown in our study. Overall, Nrf1-mediated dual regulation of proteasomes and ALP could help restore proteostasis in cells experiencing proteotoxic stress (Fig. 9).

A previous report from Goldberg and colleagues concluded that in response to proteasome inhibition, ALP genes p62/SQSTM1 and GABARAPL1 are rapidly induced to promote cell survival where p62 primarily acts by sequestering ubiquitylated proteins in inclusions (Sha et al., 2018). Although they showed that p62 is induced in an Nrf1 and NF-E2-dependent manner, the induction of GABARAPL1 and the other ALP genes together with activation of autophagy at a later time point was attributed to be Nrf1-independent. In contrast, our current study found a tight dependence of autophagy on the functional status of Nrf1. In Nrf1-depleted cells and in NGLY1-deficient cells (in which Nrf1 is inactive), using orthogonal methods, we found a clear defect in activating autophagy and the ability to clear aggresomes via aggrephagy. Furthermore, using GABARAPL1 as an example, we found direct binding of Nrf1 to its promoter region. Together with evidence from the ENCODE ChIP-seq datasets that indicate Nrf1 can bind to the regulatory regions of multiple ALP genes, our conclusions are consistent with a model in which Nrf1 can directly activate ALP genes to promote autophagy in the face of proteotoxic stress. While our manuscript was in revision, an article from Kobayashi and colleagues demonstrated that proteasome inhibitor-mediated aggrephagy proceeded via direct activation of p62 and GABARAPL1 by Nrf1 (Hatanaka et al., 2023). Also, an earlier study by Yan and colleagues demonstrated that the NGLY1-Nrf1 axis could regulate mitophagy (Yang et al., 2018). Thus, Nrf1 is shaping up to be an important proteostasis factor with the ability to govern multiple facets of cellular quality control mechanisms.

Of note, although we observed a consistent attenuation of the different aspects of ALP in Nrf1^KO^ and NGLY1^KO^ cells after proteasome inhibition, it was never a complete abolition of these effects. It is possible that there could be other Nrf1-independent pathway(s) operative in this context to regulate and strengthen the extent of autophagy in response to proteotoxic stress. On the flip side, our demonstration that overexpression of Nrf1 by itself could mobilize ALP even in the absence of proteasome inhibition is significant and underscores the importance of this transcription factor in this context.

Besides the mechanistic aspects, our findings also have important translational implications. In cancer therapies that utilize proteasome inhibitors (e.g., multiple myeloma), there is now a stronger justification to inhibit the Nrf1 pathway to increase the treatment efficacy. Although transcription factors such as Nrf1 remain undruggable directly, given the complexity of the pathway, there are ample opportunities to target activating enzymes such as the ATPase p97/VCP, N-glycanase NGLY1, and the protease DDI2, all of which are essential for the functional maturation of Nrf1 (Northrop et al., 2020). On the other hand, in certain neurodegenerative diseases (e.g., Alzheimer’s disease) that exhibit ubiquitylated protein aggregates in the neurons, there could be interest in enhancing the Nrf1-dependent proteasome and autophagy pathways to clear the aggregates and improve proteostasis.

## Materials and methods

### Plasmid constructs

Nrf1-LentiCRISPRv2 was previously described (Vangala et al., 2016). To generate pLPCX-HA-Nrf1-3xFlag (a construct expressing p120 with N-terminal HA tag and C-terminal 3xFlag), we amplified Nrf1 ORF with HindIII/EcoRI-containing primers and cloned it into pLPCXpuro. Similarly, we generated pLPCX-HA-p110Nrf1-3xFlag (a construct expressing p110, the active form of Nrf1), which is devoid of the N-terminal 104 amino acid of p120. These constructs are referred to as p120 and p110, respectively, in this paper. pBABE-puro-mCherry-EGFP-LC3B (plasmid# 22418; Addgene) was a gift from Dr. Jayanta Debnath (University of California, San Francisco, San Francisco, CA, USA). Human SQSTM1/p62 plasmid was from Horizon Discovery/Dharmacon (Cat. No OHS6085-213579813). pCSII-MCS-EF-GABARAPL1 and pCSII-MCS-EF control lentiviral vectors (Grunwald et al., 2020) were gifts from Dr. Do-Hyung Kim (University of Minnesota, Minneapolis, MN, USA).

### Cell culture

NIH-3T3 (CRL-1658; ATCC), HT22 (SCC-129; Sigma-Aldrich), MDA-MB-231 (HTB-26; ATCC), MEF (a gift from Dr. Tadashi Suzuki, RIKEN, Kanagawa, Japan), HEK293T (CRL-3216; ATCC) cells, and their derivatives were grown in Dulbecco’s Modified Eagle Medium (DMEM) (Gibco) with 10% fetal bovine serum (FBS; Atlanta Biologicals), 1X penicillin-streptomycin (Pen/Strep, Invitrogen) and 5 µg/ml Plasmocin Prophylactic (PP, InvivoGen). SH-SY5Y cells (CRL-2266; ATCC) and their derivatives were grown in DMEM:Nutrient Mixture F12 with 10% FBS, 1X Pen/Strep, and 5 µg/ml PP. All cells were maintained at 37°C in a humidified incubator with 5% CO_2_.

### Viral transduction and generation of stable cell lines

To generate viral particles, 5.3 million HEK293T cells were plated in 10-cm plates the day before transfection. Retroviral transfer plasmid (4 µg), pUMVC (3.6 µg), and pCMV-VSV-G (0.4 µg) were transfected into the cells with Lipofectamine 3000 (Cat No:L3000015; Thermo Fisher Scientific), according to manufacturer’s instructions. To generate lentiviral particles, lentiviral transfer plasmid (4 µg), pCAG-HIVgp (2 µg), pHDM-G (1 µg), and pCAG4-RTR2 (1 µg) were used. Media supernatant containing viral particles was collected at 48 and 72 h after transfection and then precipitated with PEG-it solution (Systems Bio). Concentrated viral particles were aliquoted and stored at −80°C.

The day before infection, HT22 cells were plated in either six-well plates or 10-cm plates at around 60% confluency. To generate stable cells for overexpression or for knock-out, one aliquot of viral particles with freshly added polybrene (4 µg/ml) was used to infect cells plated in a six-well plate in serum-free media, which was replaced with complete media the next day. HT22 and NIH-3T3 cells that were infected with mCherry-GFP-LC3B virus were selected by FACS, 2 days after infection. Cells that were infected with Nrf1-LentiCRISPRv2 virus were selected with 1 µg/ml puromycin for 2 wk.

Nrf1-depleted cells in 10-cm plates were first infected with two aliquots of p120 viral particles. Three days after infection, cells were split and infected again with two aliquots of viral particles for an efficient rescue. These cells were used to perform experiments after the second infection.

### Quantitative reverse transcription PCR (qRT-PCR)

Cells were plated in six-well plates to reach 70% confluency at the time of treatment. Cells were treated with either 200 nM CFZ or 200 nM BTZ for 6 and 24 h. At the end of treatment, cells were washed with PBS once and collected by scraping the cells in 1 ml PBS. They were centrifuged at 14,000 rpm for 1 min at 4°C, PBS was aspirated off, and cell pellets were stored at −80°C. RNA isolation was performed by RNeasy Kit (Qiagen) with the addition of DNAse (Qiagen) treatment. 1 µg of RNA was used to make cDNA with iScript Reverse Transcriptase (Bio-Rad). qRT-PCR reactions were prepared with iTaq Universal SYBR green supermix (Bio-Rad) and ran in C1000 Touch Thermal Cycler (Bio-Rad). Depending on experimental conditions, either 18S or GAPDH was used as an internal control for normalization. Data were analyzed using CFX Manager 3.1 (Bio-Rad). The primers used for qRT-PCR are listed in Table S2. Calculation of the P value was done by Student’s *t* test.

### Immunoblot analysis

Cells were plated in six-well plates to reach 70% confluency at the time of treatment. Cells were washed once with ice-cold PBS and then scraped in RIPA Buffer (20 mM Tris-HCl pH 7.4, 1% sodium deoxycholate, 1% Triton X-100, 0.1% SDS, 150 mM NaCl, and 1 mM EDTA) containing protease and phosphatase Inhibitor cocktail (Cat No:PI78447; Thermo Fisher Scientific) or collected in 4X Laemmli protein sample buffer on ice. Lysates were then sonicated at a low power setting for 5 s once, then incubated on ice for 20 min, and centrifuged at 14,000 rpm for 20 min at 4°C. Protein concentrations were measured by Bradford assay. Samples for Western blots were prepared with 4X Laemmli protein sample buffer (Cat No:1610747; Bio-Rad) with the addition of 2-mercaptoethanol on the day of lysis, followed by boiling for 7 min. 20 µg of protein was used for SDS-PAGE, and for fluorescent Western blotting, gels were transferred onto Immobilon FL PVDF membranes (Cat No:IPFL00005; Thermo Fisher Scientific); for enhanced chemiluminescence (ECL) Western blotting, gels were transferred onto Immobilon P PVDF membrane (Cat No:IPVH00010; Thermo Fisher Scientific). For fluorescent Western blotting, membranes were blocked with Intercept TBS blocking buffer (Cat No:NC1660550; Thermo Fisher Scientific) for 1 h, and for ECL Western blotting, membranes were blocked with 5% milk in TBST for 1 h.

Blots were incubated overnight with specific primary antibodies, followed by incubation with secondary antibodies for 1 h at room temperature. The antibodies used were LC3B (Cat No:2775S; Cell Signaling) at 1:1,000, β-actin (Cat No:A5441; Millipore Sigma) at 1:10,000, Nrf1 (Cat No:8052S; Cell Signaling) at 1:1,000, Cathepsin D (Cat No:69854S; Cell Signaling) at 1:500, ATG4A (Cat No:7613S; Cell Signaling), SQSTM1/p62 (Cat No:5114S; Cell Signaling), VPS37A (Cat No:11870-1-AP; Protein Tech), Ubiquitin (Cat No:BML-PW0930 & ADI-SPA-203; Enzo) at 1:1,000, α/β-Tubulin (Cat No:2148S; Cell Signaling) at 1:5,000, and GABARAPL1 (Cat No:26632S; Cell Signaling) at 1:1,000. IRDye 800CW Goat-anti-Rabbit antibody (Cat No:102673-330; VWR) and IRDye 680RD Goat-anti-Mouse antibody (Cat No:102673-408; VWR) at 1:20,000 dilutions. After secondary antibody incubation, membranes were washed with TBST (four times, 5 min each) and fluorescent Western blots were dried for 1 h in the dark before imaging. Both fluorescent and ECL Western blots were imaged with the LiCOR Odyssey Fc Imaging System. Quantification of Western blot signals was done by Image Studio Lite V5.2 software and Student’s *t* test was used to determine P values. All graphs were plotted by using GraphPad Prism software.

### LC3B puncta analysis

0.25 million HT22 mCherry-GFP-LC3B cells were plated in six-well plates with coverslips. Cells were treated with either DMSO or 200 nM BTZ for 18 h. After treatment cells were washed with PBS 1X and fixed with ice-cold methanol for 10 min, followed by PBS wash 3X and mounted on slides with Prolong Gold Antifade Mountant with DAPI. Images were captured using spinning disc confocal microscopy with 63× objective. The GFP and RFP (mCherry) signals were imaged using a line sequential scan setting with excitation laser lines at 488 and 543 nm, respectively. The emission signals were collected at 495–530 nm (GFP, channel 1) and 590–650 nm (RFP, channel 2). Puncta staining was quantified by ImageJ software with the help of “Green and Red Puncta Colocalization” macro developed by Daniel J. Shiwarski, Ruben K. Dagda, and Charleen T. Chu. HT22 mCherry-GFP-LC3B Nrf1^KO^ and Nrf1^KO^ with p62 overexpression cells were imaged with a Zeiss AxioImager Z2 with 63× objective. The software used for image acquisition was the Neurolucida 2023 software. ImageJ was used for puncta quantification for the p62 assays. Images were set to 8bit, a threshold was created to get rid of the background, and images were set to binary. This was done on each image for GFP and mCherry, while the Dapi section was used to determine the number of cells, which was used for normalization. Particle analysis was used to determine the number of green and red puncta. For NIH-3T3 mCherry-GFP-LC3B cells were plated in glass-bottom 96-well plates, and after fixation, images were acquired using a high-content microscope (Operetta, PerkinElmer). Nine fields per well were captured and the images were analyzed using the manufacturer’s software.

### Aggresome assays

0.25 million HT22 and 0.4 million MEF cells were treated with either 50 nM CFZ or with DMSO for 20 h on coverslips in six-well plates. The cells that received a recovery period after a 20 h CFZ treatment were washed with PBS twice and then supplied with fresh complete media for 20 h. At the end of treatment, cells were washed with PBS, fixed with ice-cold methanol, blocked with blocking solution (1% BSA and 0.3% Triton-X in PBS), and stained with FK2 antibody at a 1:500 dilution (Cayman Chemicals; Milipore) for overnight, followed by washing and incubation with Goat-anti-Mouse Alexa-flour 555 (Invitrogen) for 1 h. After incubation, cells were washed once in blocking solution and then incubated for 5 min in Hoechst diluted 1:10,000 in PBS. Hoechst was aspirated off followed by one wash with PBS and two with deionized water before coverslips were mounted. Spinning disc confocal microscopy with 63× objective was used to image the cells. ImageJ with Aggrecount macro was used to quantify images (Klickstein et al., 2020; Mukkavalli et al., 2021).

For the biochemical version of the aggresome assays, after the termination of treatments, cells were pelleted down at 800 *g* for 3 min, followed by washing 1× with PBS. Pellets were lysed in NP-40 lysis buffer (1% NP-40, 125 mM Tris-HCl pH 7.2, 150 mM NaCl, 1 mM EDTA, protease and phosphatase inhibitor cocktail) for 10 min on ice, and centrifuged for 15 min at 16,000 *g*. Supernatants (soluble fractions) were discarded. Pellets were washed once with NP-40 lysis buffer and centrifuged to remove the residual soluble fractions. SDS buffer (2% SDS, 62.5 mM Tris-HCl pH 6.8, 10% Glycerol, 50 mM DTT, protease and phosphatase inhibitor cocktail) was added to the pellet, sonicated (10–15 power; 5 s pulses 3×). The supernatant was collected after centrifugation at 16,000 *g* for 10 min as an insoluble fraction (aggregates). This fraction was analyzed using immunoblotting.

### Chromatin immunoprecipitation (ChIP)

ChIP assay was performed as described previously (Vangala and Radhakrishnan, 2019). Briefly, cells were crosslinked for 10 min with 1% (wt/vol) formaldehyde, followed by quenching with 0.125 M glycine. After cold PBS washes 2X, pellets were collected at 800 *g* at 4°C. Chromatin was isolated using EZ-Magna ChIP A/G (Millipore) kit according to the manufacturer’s protocol. Covaris M220 was used with 10% duty factor (df) for 12 min for chromatin shearing. The supernatant was collected from sheared chromatin at 10,000 *g* for 10 min and precleared with 20 μl of protein A/G magnetic beads for 1 h at 4°C. Immunoprecipitation was completed with 5 μg of specific antibody for 50 μl of precleared chromatin overnight at 4°C. After immunoprecipitation, beads were consequently washed with a series of buffers and eluted in elution buffer according to the manufacturer’s protocol. Qiagen columns were used for the purification of eluants. qPCR was used to analyze the chromatin pulldown using specific primers shown in Table S2.

### Cloning of the promoter and luciferase assays

Promoter regions of ∼1.0 kb of human GABARAPL1, CTSD, and VPS37A upstream of +1 TSS were synthesized (GenScript) and cloned into pGL3-basic luciferase vector (Cat. No E1751; Promega) using KpnI & HindIII restriction sites. Putative ARE sites (TGAnnnnGC) were mutated to create all mutant promoters. To determine the promoter activity, HEK293T cells were transfected with the promoter-luciferase constructs, pRL-TK (Cat. No. E2241; Promega) alone or in combination with Nrf1-p110 in a ratio of 3:1:6 using Lipofectamine 3000. The total plasmid concentration was kept to 2 μg using the control plasmid. Transfected cells were treated with proteasome inhibitor overnight after 48 h of transfection. Luciferase assay was carried out using Dual-Glo Luciferase Reporter Assay System (E2920; Promega) according to the manufacturer’s protocol. Relative luciferase values were calculated after normalizing the firefly with renilla luciferase activities.

### Transmission electron microscopy (TEM)

NIH-3T3 cells were treated for 20 h with 200 nM CFZ, washed with PBS, and then fixed with 2.0–2.5% glutaraldehyde in 0.1 M cacodylate buffer at room temperature for 1 h. Cells were then stored at 4°C in the fixative until dehydration. Cells were dehydrated in graded acetone series: 50%, 70%, 80%, and 95% for 10 min each and then 100% acetone three times for 10–15 min. After dehydration, they were infiltrated with a 50/50 mix of acetone and EMbed 812 resin (Polysciences, Inc.) mix overnight. Then the cells were infiltrated with pure EMBed 812 resin for 8 h and then again overnight. The cells were then placed in an oven at 55–60°C for 2 days. They were then sectioned on a Leica EM UC7 ultramicrotome (Leica Microsystems) at 80–100 nm thick sections on grids and stained with 5% Uranyl Acetate and Reynold’s Lead Citrate. Sections were imaged using a JEOL JEM 1400Plus TEM (JEOL USA, Inc.) with the Gatan One View digital camera (Gatan Inc.), which was kept at −5°C. The software used for image acquisition was the Digital Micrograph version 3.3. In the electron micrographs, AVs (autophagosomes and autophagolysosomes) were identified using previously established criteria (Dunn, 1990; Nixon et al., 2005). The analysis was done blinded to the samples. Quantitative morphometric analysis of the number, area, and total area occupied by AVs per cytoplasmic area was calculated using ImageJ (NIH).

### Statistical analysis

Data are presented as mean ± SD of at least three experiments. We used either two-way ANOVA or a Student’s *t* test when appropriate. All statistical analyses were conducted using GraphPad Prism 8, and P values <0.05 were considered significant.

### Online supplemental material

Fig. S1 shows Nrf1-mediated regulation of autophagy–lysosome pathway (ALP) genes upon proteasome inhibition in SH-SY5Y, HT22, and MDA-MB-231 cells. Fig. S2 shows adding back Nrf1 in Nrf1-deficient cells rescues suppressed expression of ALP genes upon proteasome inhibition in SH-SY5Y-Nrf1^KD^ and HT22-Nrf1^KO^ cells. Fig. S3 shows Nrf1^KO^ cells display defects in basal and starvation-induced autophagy in NIH-3T3 cells. Fig. S4 shows that p62 overexpression does not rescue aggresome clearance in Nrf1^KO^ cells. Table S1 shows Nrf1 binding locations derived from ENCODE project database (Identifier: ENCSR245QUM). Table S2 lists the primers used in quantitative RT-PCR (qRT-PCR) and chromatin immunoprecipitation (ChIP)-qPCR assays.

## Supplementary Material

Table S1 shows Nrf1 binding locations derived from ENCODE project database (Identifier: ENCSR245QUM).

Table S2 shows primers used in quantitative RT-PCR (qRT-PCR) and chromatin immunoprecipitation (ChIP)-qPCR assays.

SourceData F1 is the source file for Fig. 1.

SourceData F2 is the source file for Fig. 2.

SourceData F5 is the source file for Fig. 5.

SourceData F6 is the source file for Fig. 6.

SourceData F7 is the source file for Fig. 7.

SourceData F8 is the source file for Fig. 8.

SourceData FS4 is the source file for Fig. S4.

## Data Availability

The data are available from the corresponding author upon reasonable request.
