# Supplementary figures and images for: Transcription factor Nrf1 regulates proteotoxic stress-induced autophagy
Source: J Cell Biol. 2024 Apr 24;223(6):e202306150. doi: 10.1083/jcb.202306150 (PMC11040505; doi:10.1083/jcb.202306150)

Source Data for Figure 1C

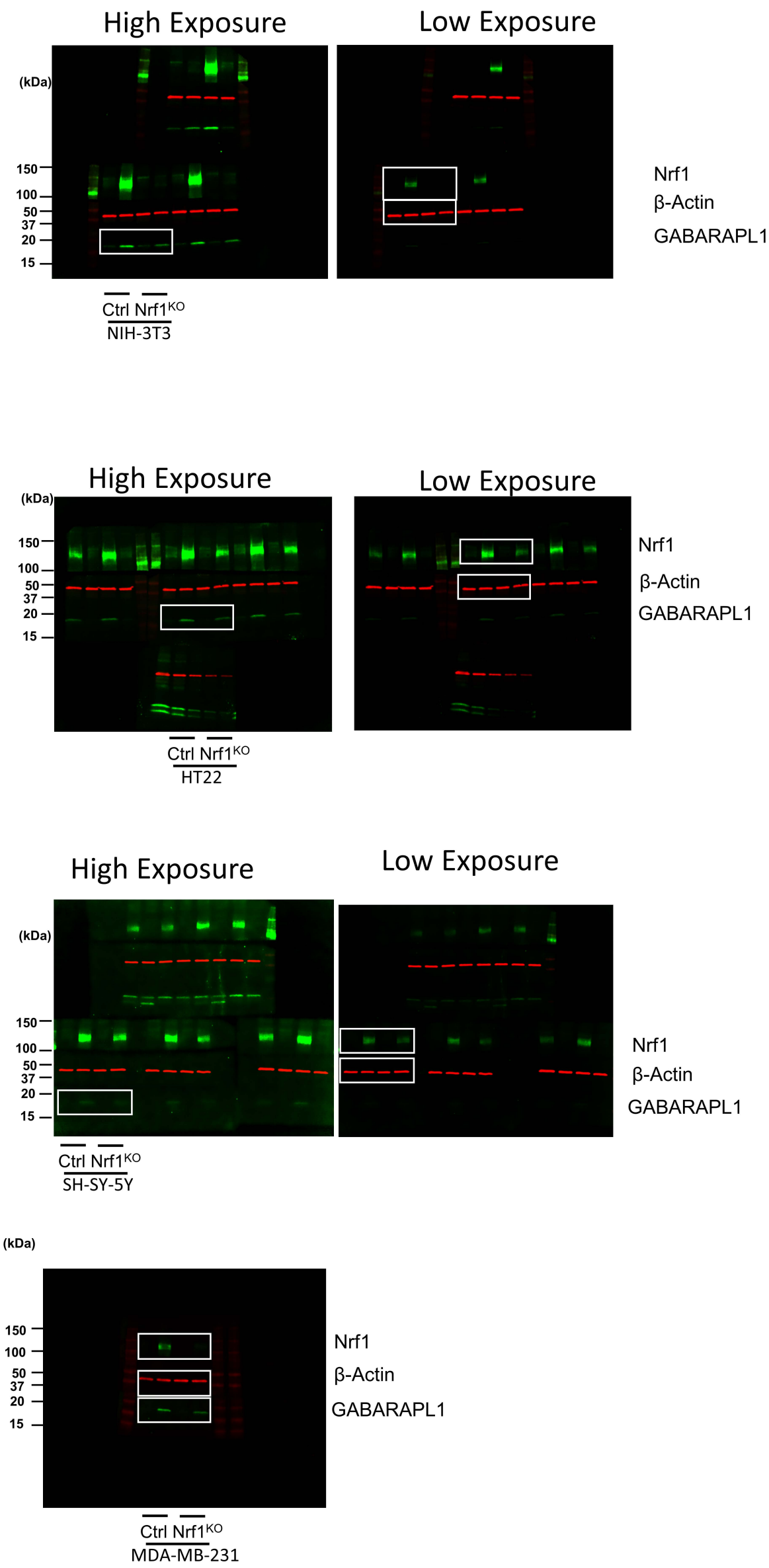

Source Data for Figure 1E

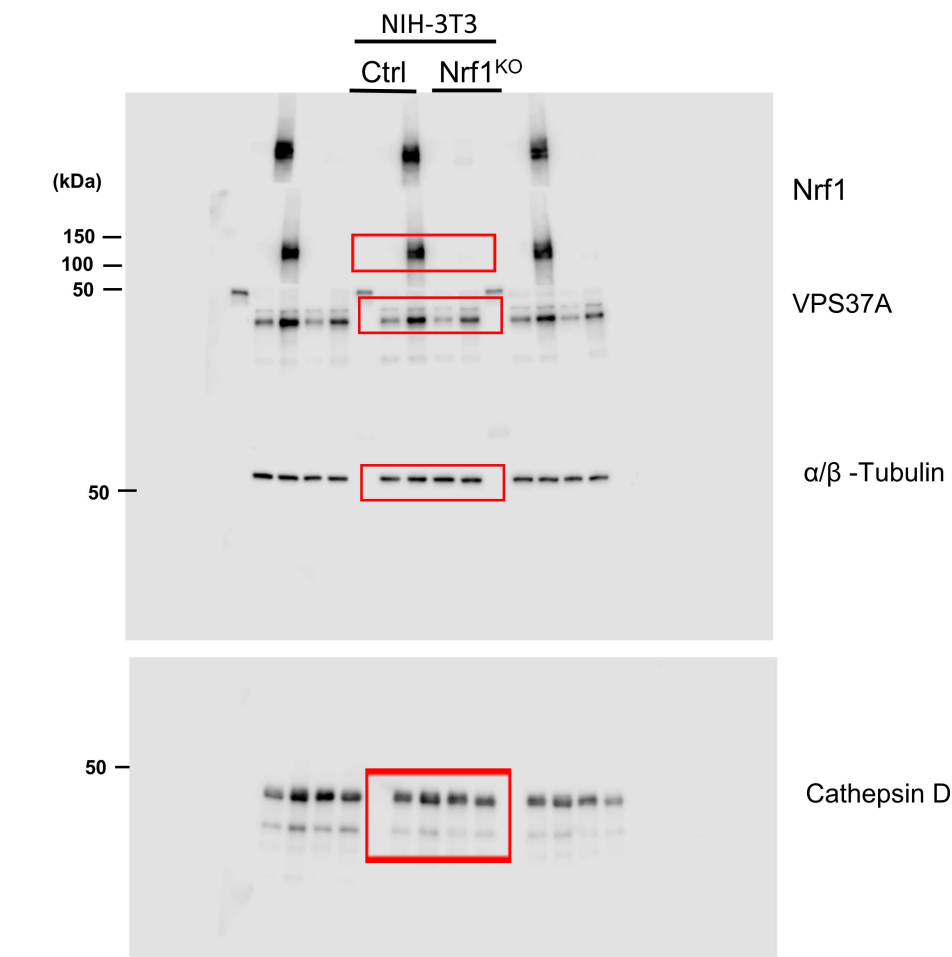

High Exposure

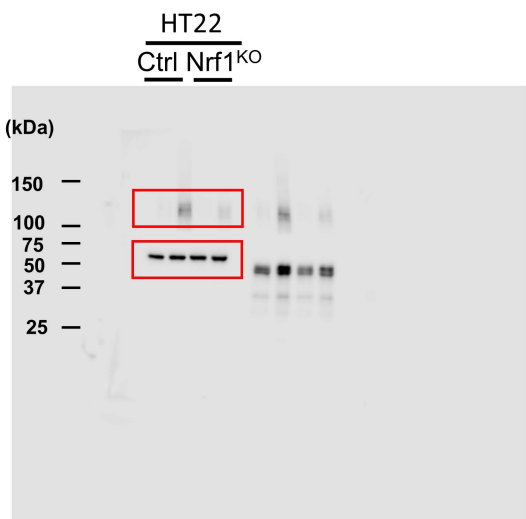

Low Exposure

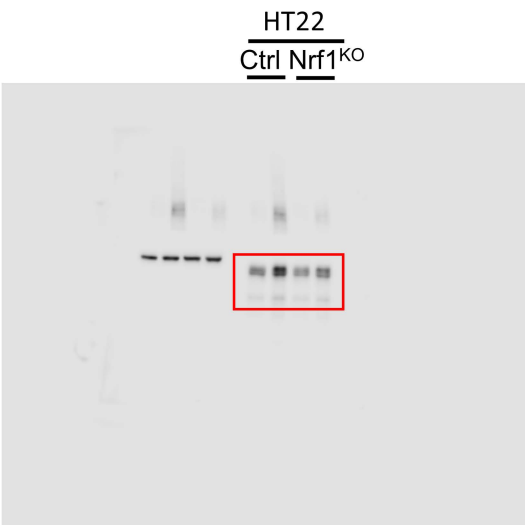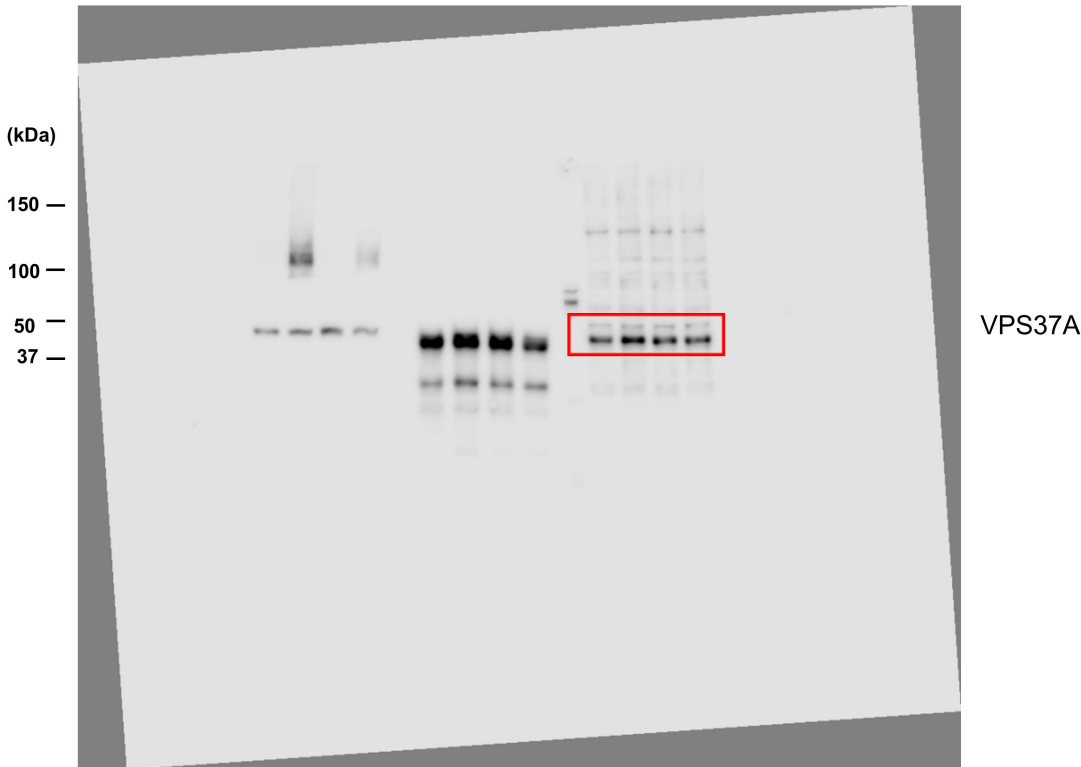

Supplement: SourceData F1 — is the source file for Fig. 1. [file JCB_202306150_SourceDataF1.pdf]

Source Data for Figure 2B

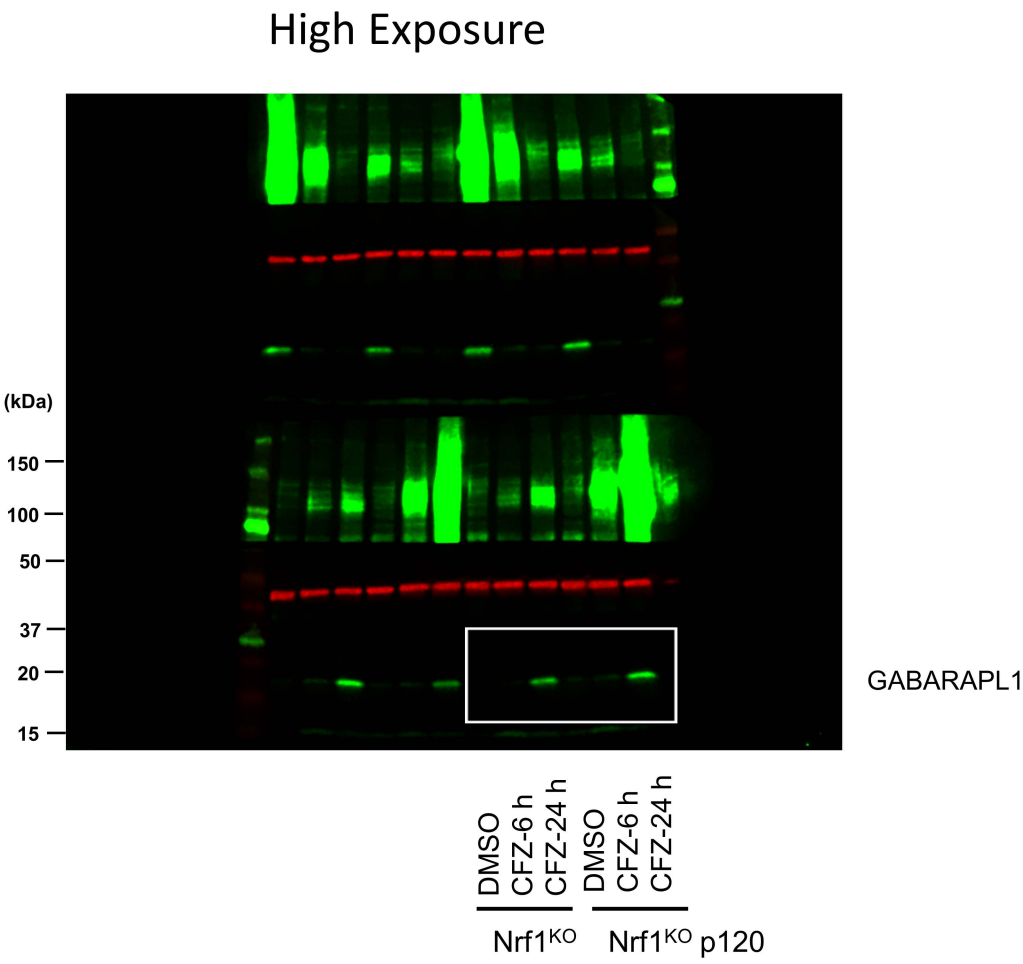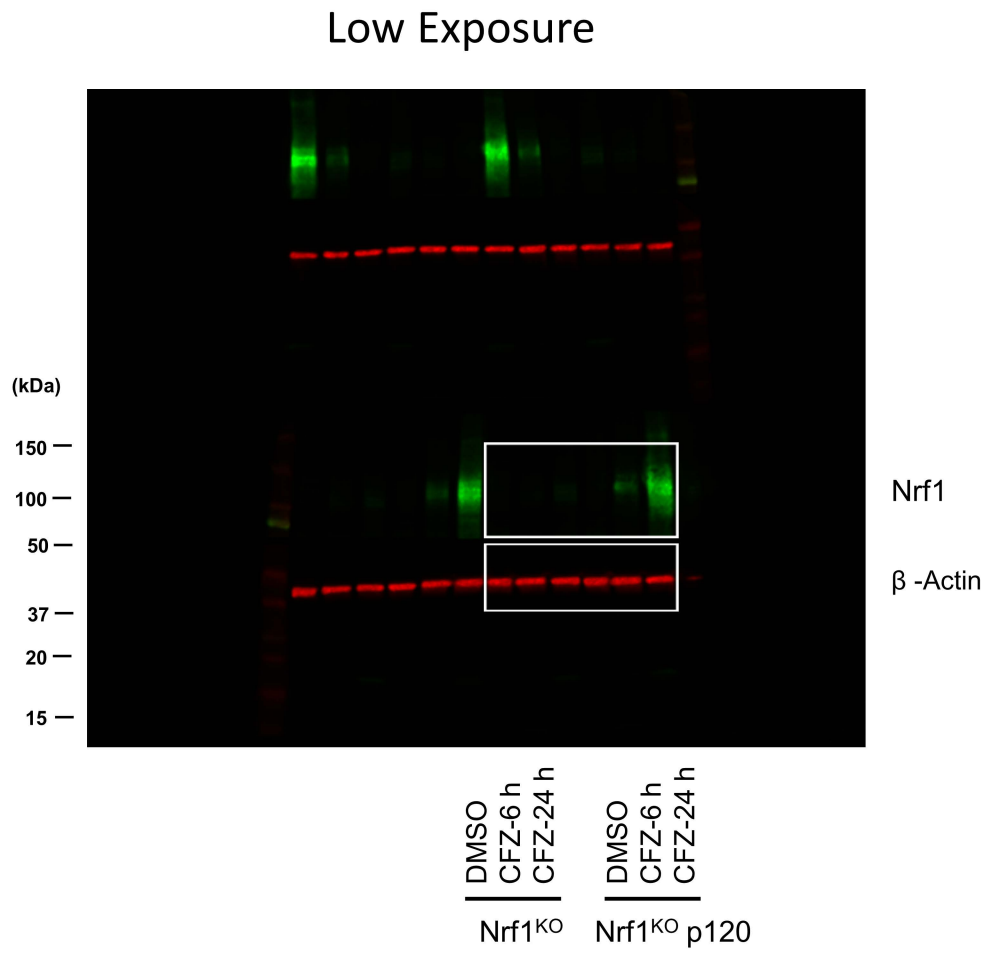

Source Data for Figure 2D

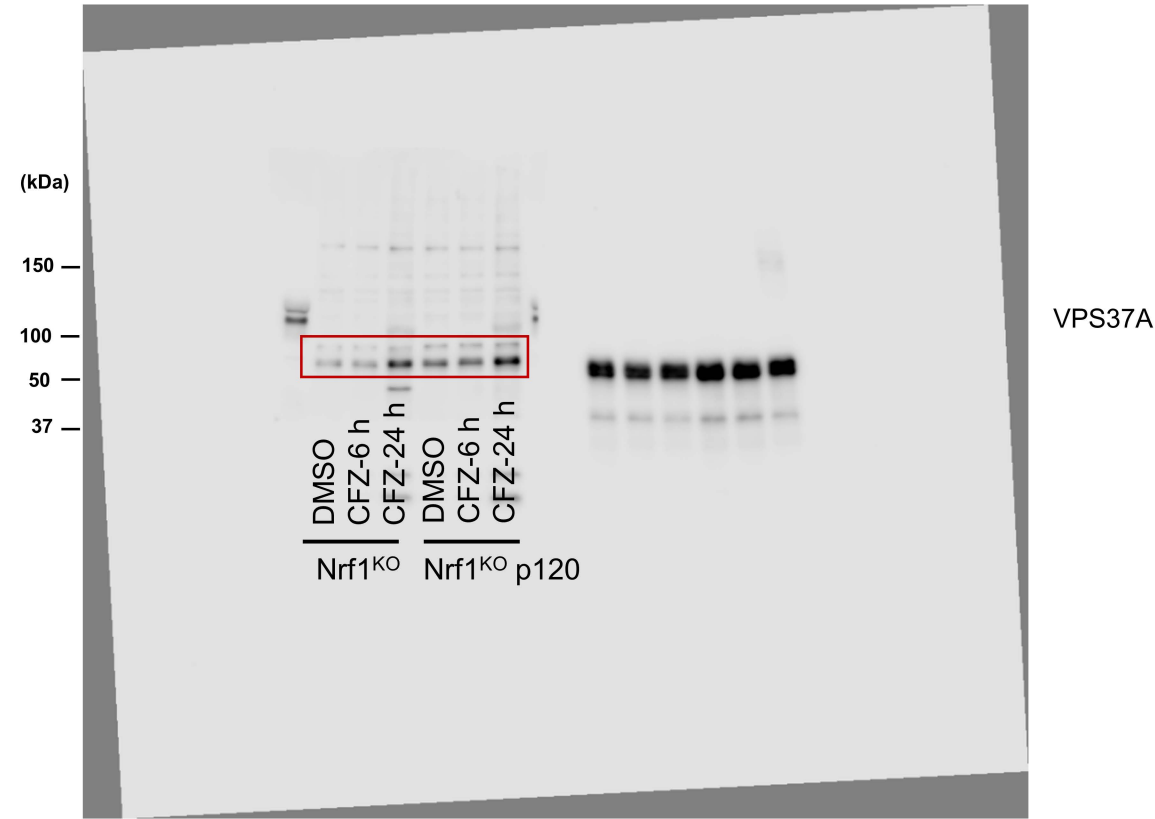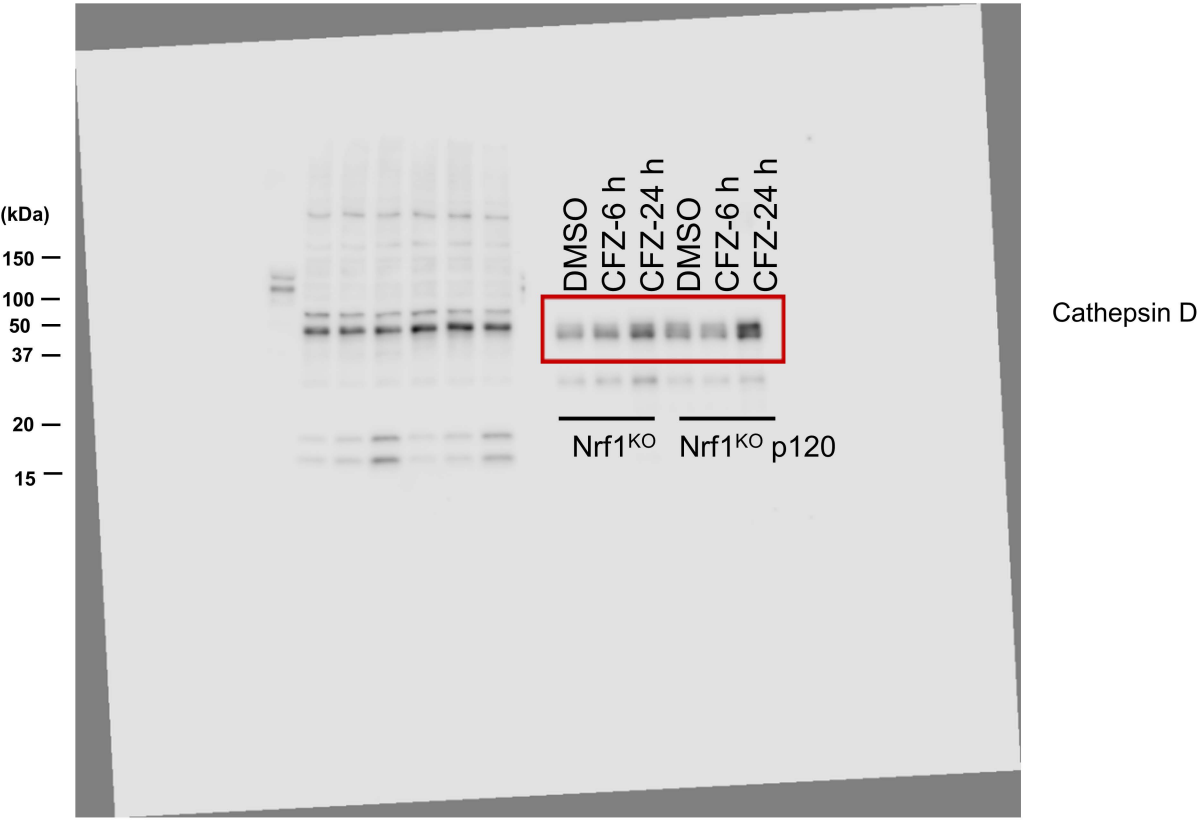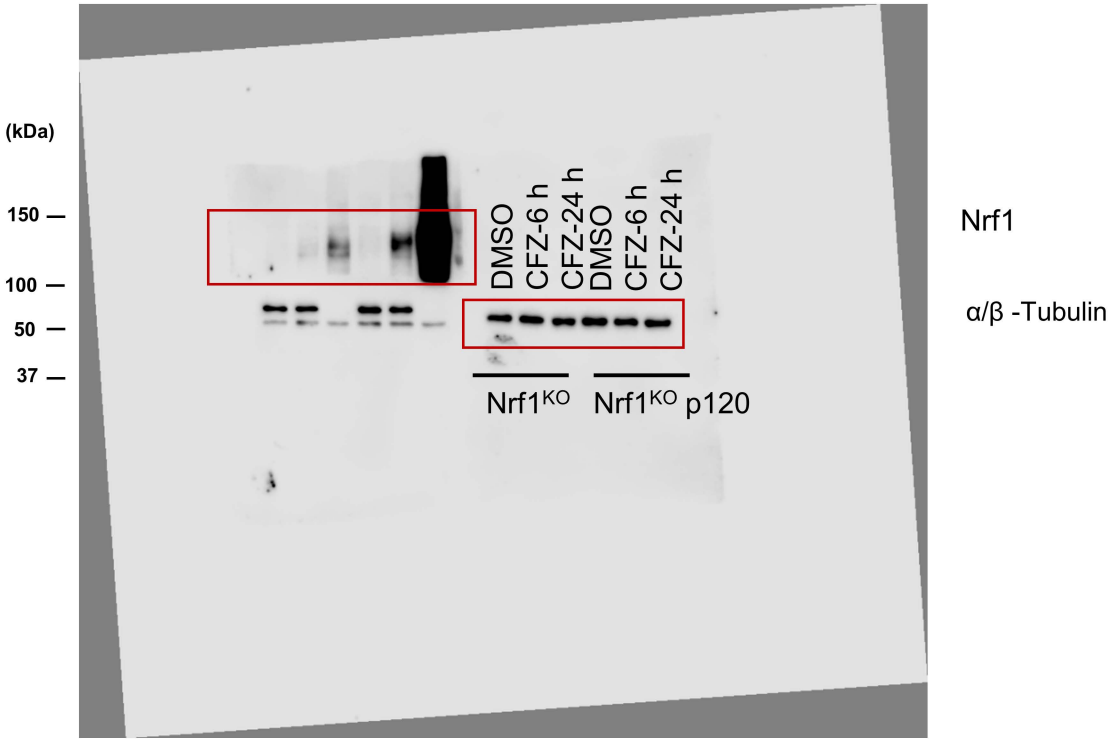

Supplement: SourceData F2 — is the source file for Fig. 2. [file JCB_202306150_SourceDataF2.pdf]

Source Data for Figure 5D

Low Exposure

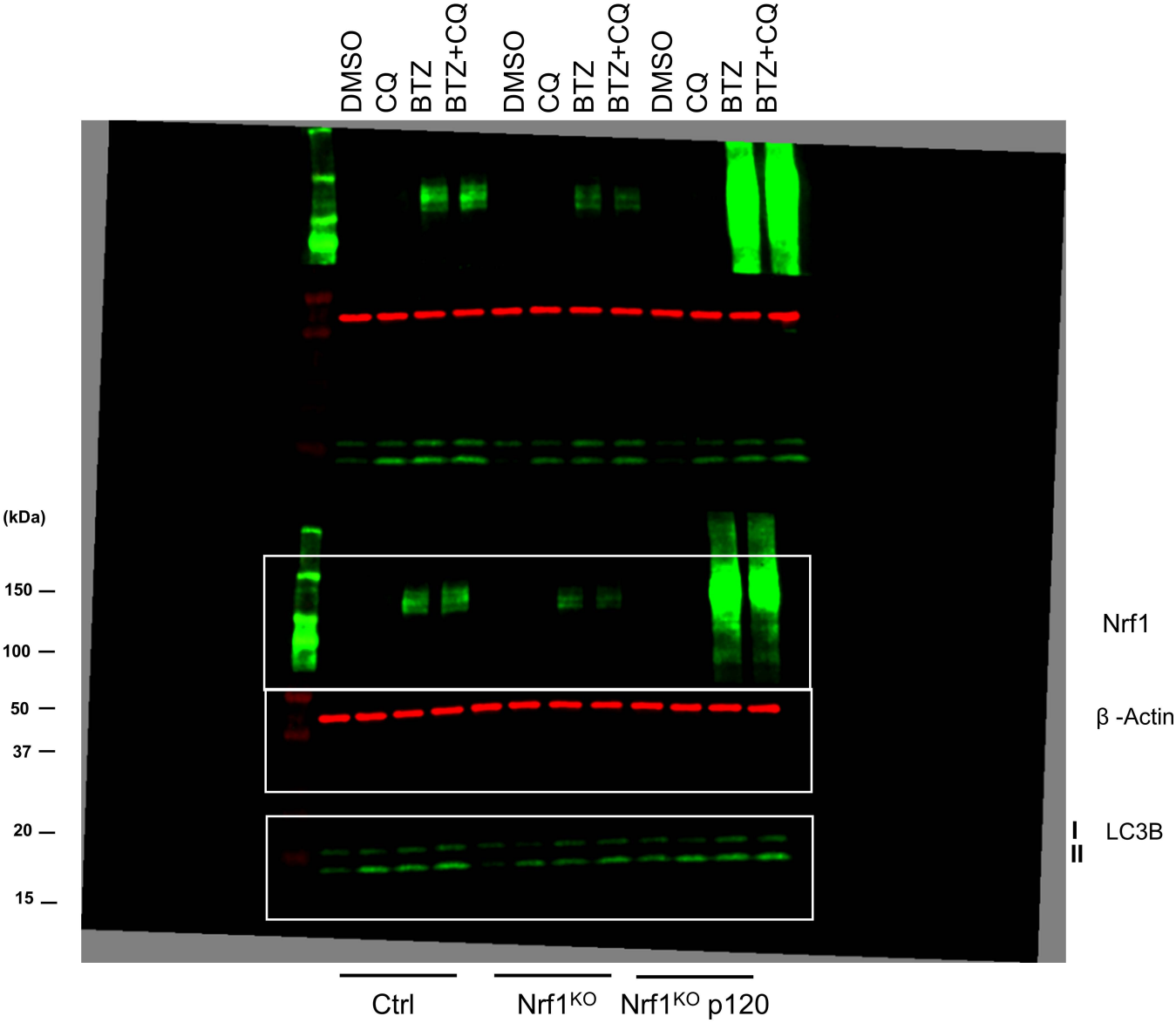

High Exposure

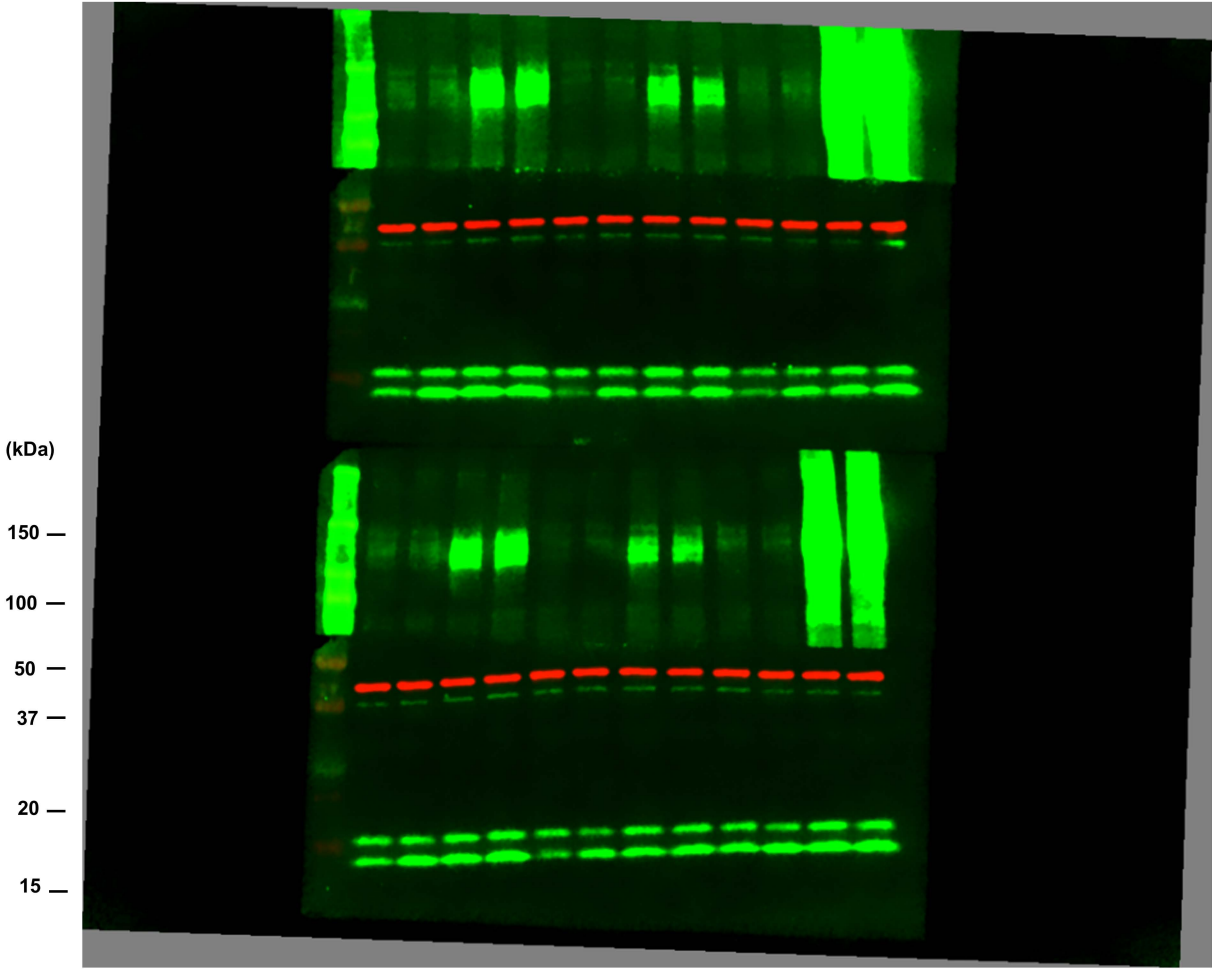

Supplement: SourceData F5 — is the source file for Fig. 5. [file JCB_202306150_SourceDataF5.pdf]

Source Data for Figure 6D

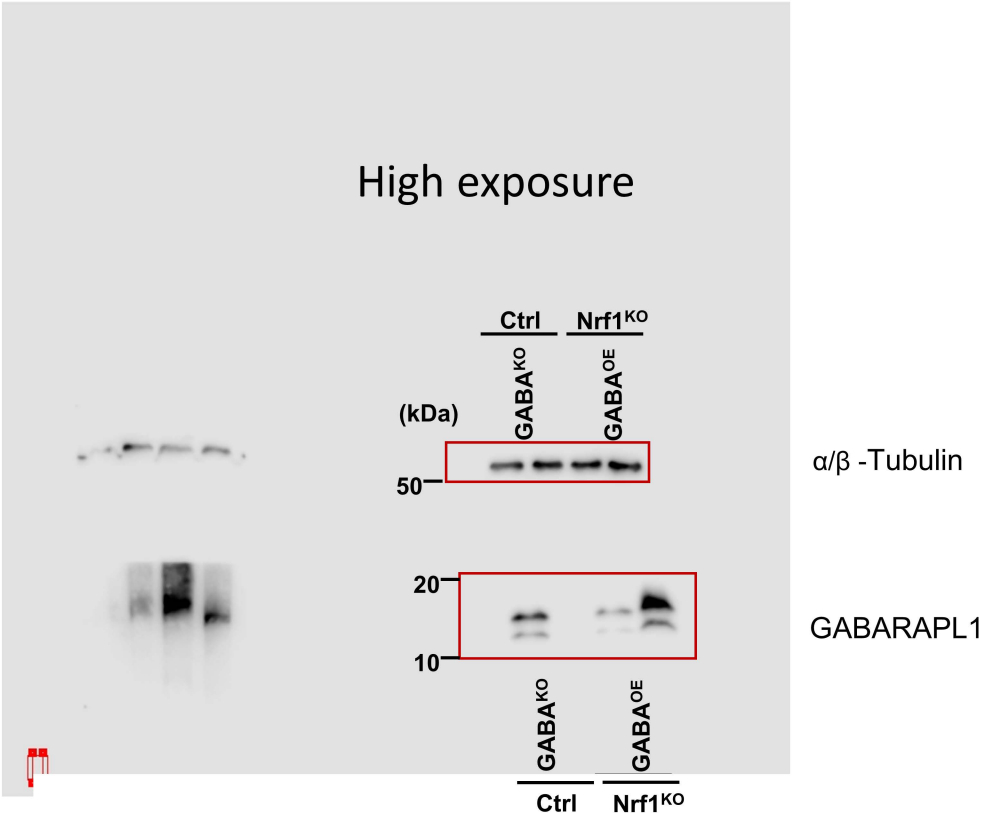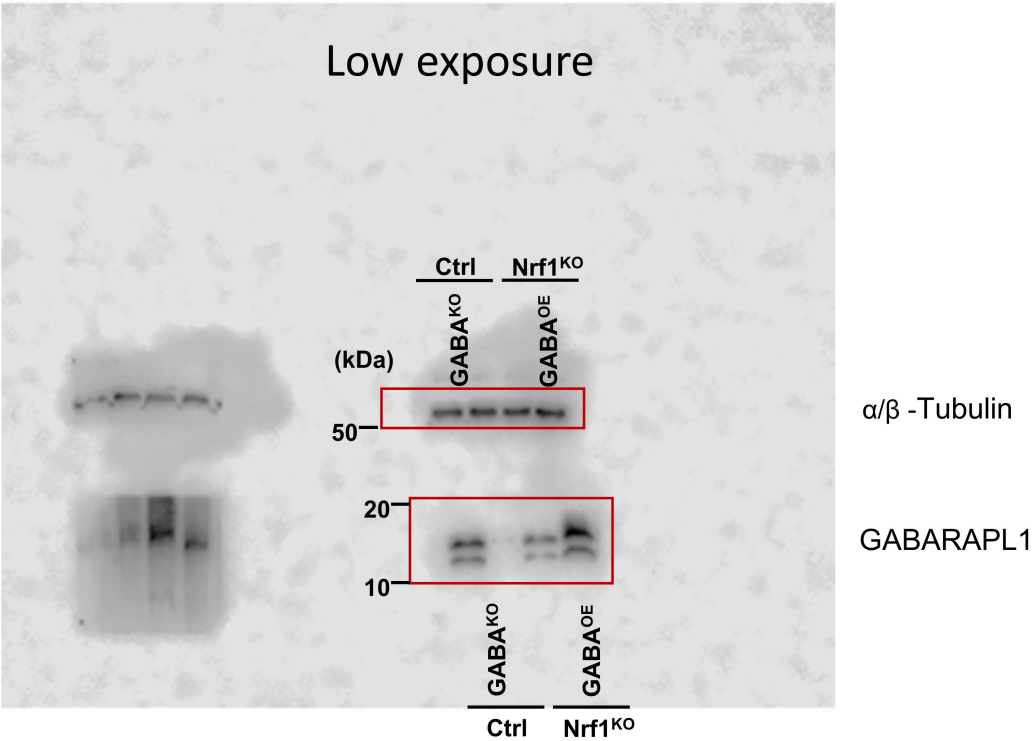

Source Data for Figure 6E

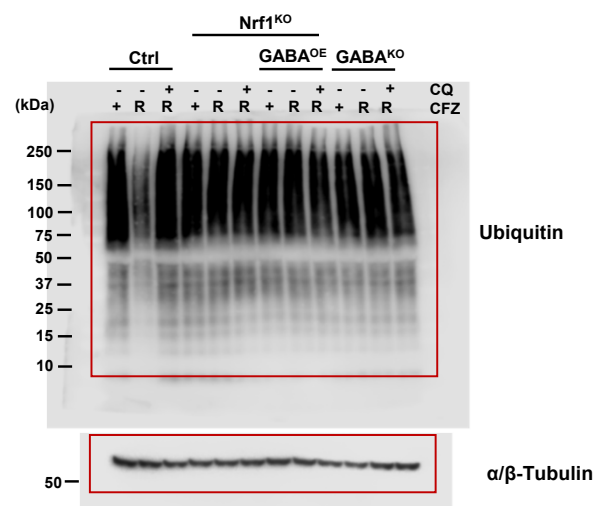

Supplement: SourceData F6 — is the source file for Fig. 6. [file JCB_202306150_SourceDataF6.pdf]

Source Data for Figure 7B

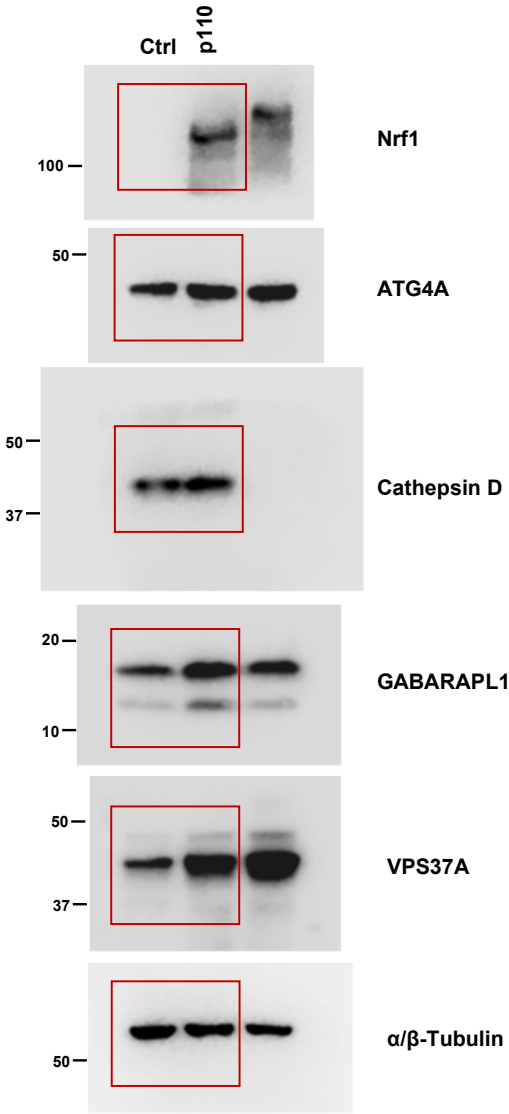

Source Data for Figure 7E

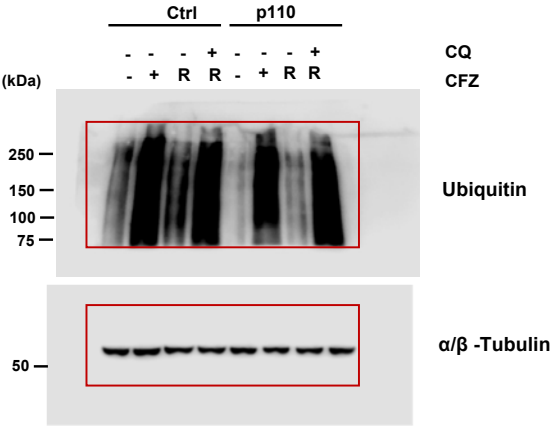

Supplement: SourceData F7 — is the source file for Fig. 7. [file JCB_202306150_SourceDataF7.pdf]

Source Data for Figure S4

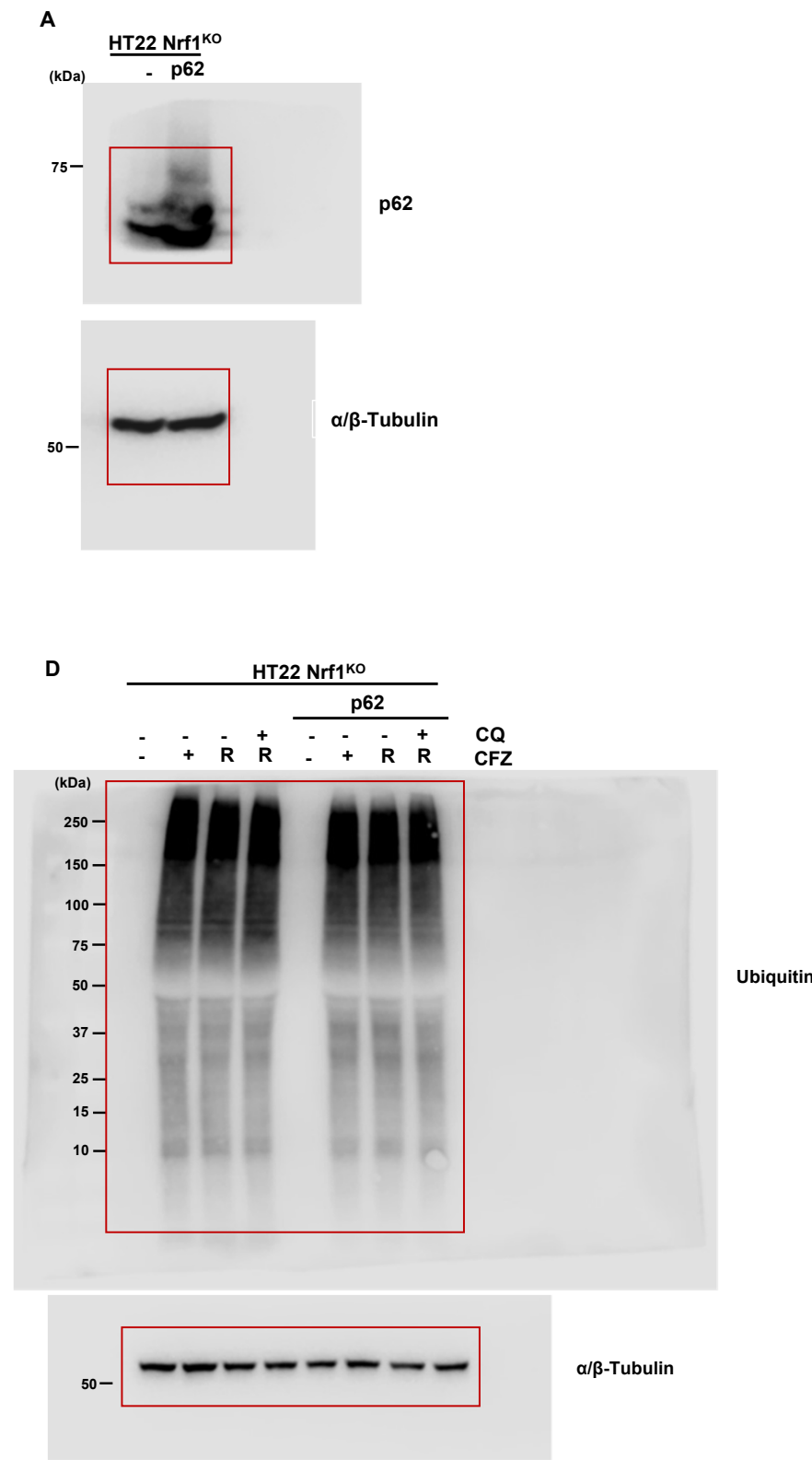

Supplement: SourceData FS4 — is the source file for Fig. S4. [file JCB_202306150_SourceDataFS4.pdf]
