# Supplementary material for: Transcription factor Nrf1 regulates proteotoxic stress-induced autophagy
Source: J Cell Biol. 2024 Apr 24;223(6):e202306150. doi: 10.1083/jcb.202306150 (PMC11040505; doi:10.1083/jcb.202306150)

Source Data for Figure 8C

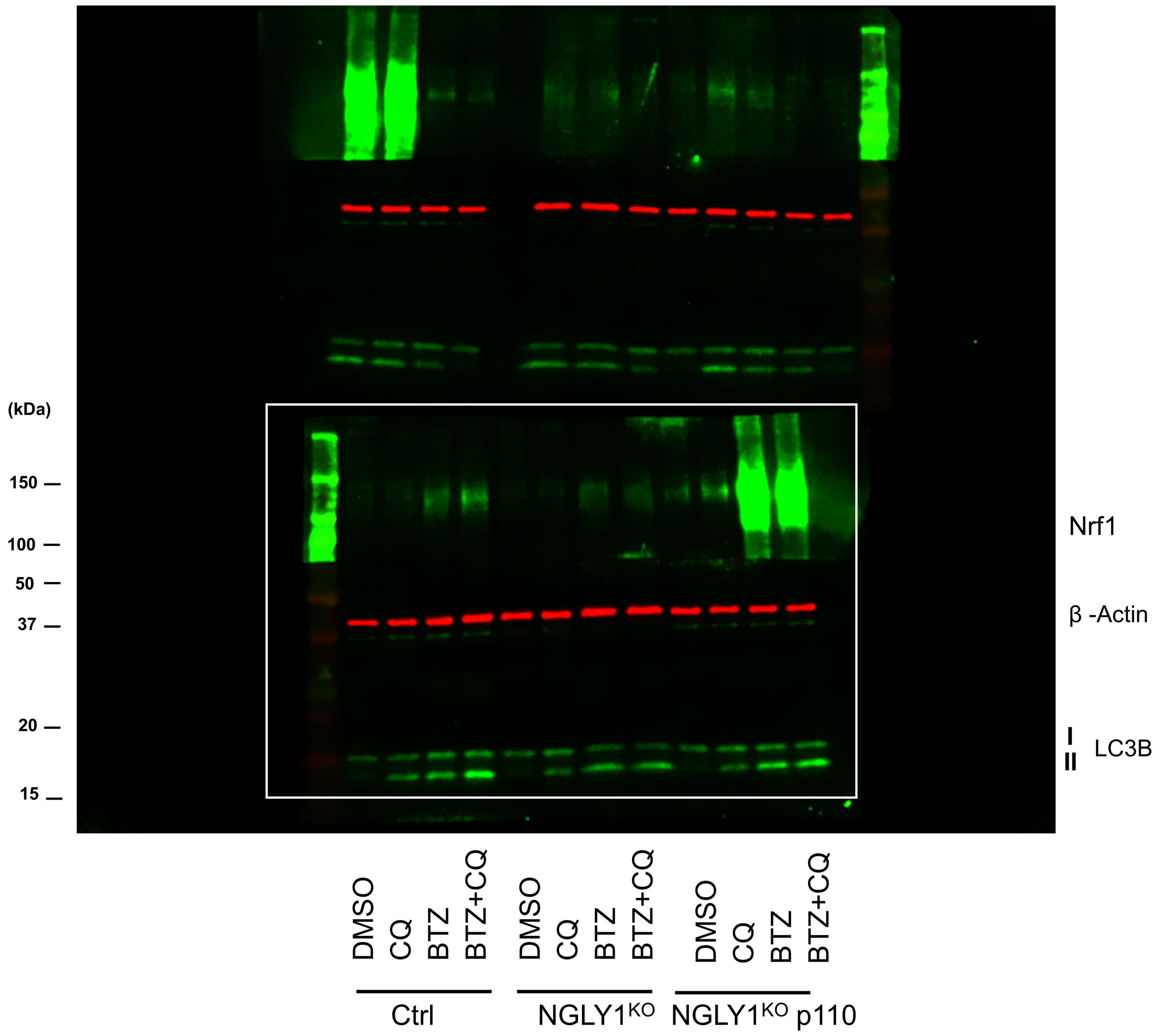

Source Data for Figure 8E

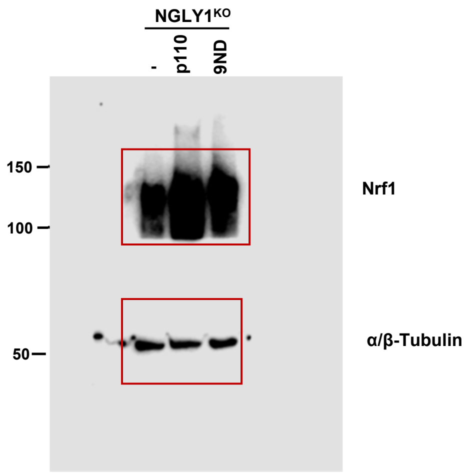

Source Data for Figure 8F

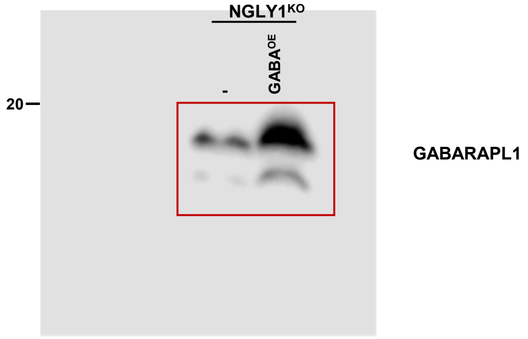

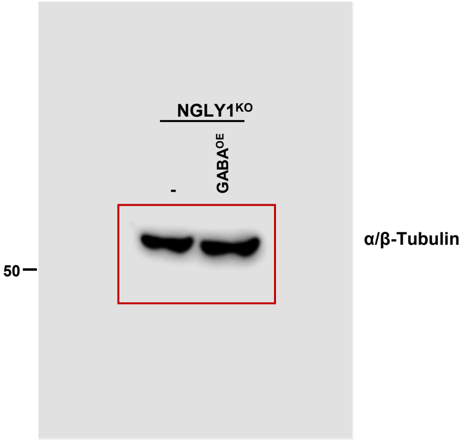

Source Data for Figure 8G

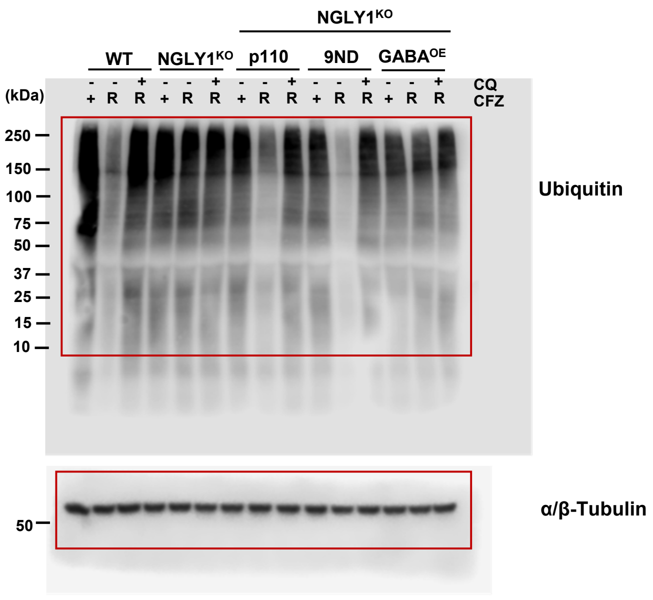

Supplement: SourceData F8 — is the source file for Fig. 8. [file JCB_202306150_SourceDataF8.pdf]
